# Deciphering the Structural Basis of Allosteric Inhibition of Mutant Epidermal Growth Factor Receptor and Identification of Novel Inhibitors

**DOI:** 10.34133/csbj.0118

**Published:** 2026-05-27

**Authors:** Sapna Pal, Debasisa Mohanty

**Affiliations:** Bioinformatics Center, BRIC-National Institute of Immunology, New Delhi 110067, India.

## Abstract

The non-small cell lung cancer-associated epidermal growth factor receptor (EGFR) mutant L858R/T790M confers resistance to first- and second-generation tyrosine kinase inhibitors (TKIs). Allosteric inhibitors have emerged as alternative therapeutic agents. Unlike orthosteric inhibitors, they preferentially stabilize EGFR in an inactive conformation. Hence, understanding the mechanistic basis of this inhibition is essential for designing potent allosteric inhibitors. In this study, we performed microsecond-scale molecular dynamics (MD) simulations on the inactive conformations of apo-EGFR^Wild^ and apo-EGFR^L858R/T790M^ to explore how cancer-associated mutations induce a conformational shift toward the active kinase state. Simulations of allosteric inhibitor (EAI001)-bound EGFR^L858R/T790M^ revealed that inhibitor binding enhances the inactive-state population by suppressing active-like excursions of the K745–E762 distance and modulating key structural elements including the αC-helix and activation loop. These findings revealed the structural/conformational basis of allosteric inhibition in EGFR^L858R/T790M^. It also emphasized the importance of MD simulations in allosteric drug design for assessing the ability of the inhibitor to enhance the population of the inactive state of the mutant EGFR. We have also standardized a virtual screening protocol involving screening of an allosteric TKI library using a structure-guided machine-learning-based protein–ligand affinity predictor scoring function and evaluated top-scoring candidates by MD simulations and the Molecular Mechanics Generalized Born Surface Area free energy calculation to identify molecules that can stabilize the inactive state of mutant EGFR^L858R/T790M^. This approach identified potential allosteric kinase inhibitors predicted to be more potent than EAI001. Overall, our results elucidate the structural/conformational basis of allosteric inhibition and highlight an MD-integrated approach for targeting dynamic allosteric sites, providing a framework for discovering next-generation modulators that can overcome TKI resistance.

## Highlights

• MD simulations reveal the structural basis of allosteric inhibition in EGFR^L858R/T790M^
• Mutant EGFR shows key shifts in αC-helix, A-loop, and P-loop toward activation
• The known allosteric inhibitor stabilizes the inactive EGFR mutant by modulating critical substructures
• Novel virtual screening workflow combines ML-based scoring with MD simulations and MM/GBSA
• Potent inhibitors are identified based on propensity to drive conformational transitions

## Graphical Abstract

**Figure:**
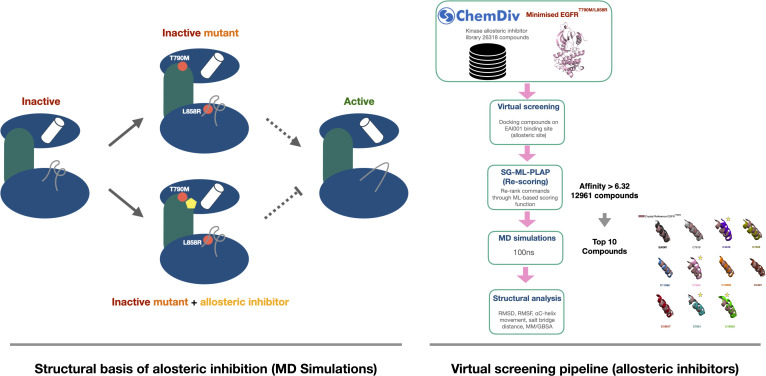

## Introduction

Epidermal growth factor receptor (EGFR) is a transmembrane glycoprotein belonging to the ErbB family, which plays a critical role in regulating cell proliferation and differentiation by activating downstream signaling pathways through phosphorylation [1]. EGFR mutations are frequently observed in various cancers, including non-small cell lung cancer (NSCLC), head and neck cancer, breast cancer, and glioblastoma [2–4]. These genomic aberrations are often associated with the continuous activation of signaling pathways, leading to uncontrolled cell proliferation and differentiation [2,5]. Given the central role of EGFR kinase in cancer progression, multiple therapeutic strategies have been developed to target its activity, including small-molecule inhibitors, monoclonal antibodies, and antisense gene therapy [6–8]. Consequently, inhibiting EGFR kinase remains a crucial area of research, particularly in the development of novel targeted therapies.

At the structural level, the structure of EGFR consists of 2 lobes (N-lobe and C-lobe) and 4 primary domains: (a) extracellular ligand-binding domain, (b) transmembrane domain, (c) intracellular kinase domain, and (d) C-terminal regulatory domain. Beyond acting as a membrane anchor, the transmembrane (TM) helix of EGFR is now recognized as an active signaling element, forming specific homo- and heterodimer packing arrangements that are coupled to receptor activation. Distinct TM dimer conformations have been correlated with inactive and active receptor tyrosine kinase states in recent structural and simulation studies [9,10]. While this work focuses on the isolated kinase domain for computational tractability, it is important to note that TM-mediated dimerization provides an additional regulatory layer.

The activation of EGFR is initiated by the binding of a ligand, such as epidermal growth factor (EGF), to the extracellular domain. This ligand-binding event triggers a series of allosteric changes in the intracellular kinase domain, which subsequently induces EGFR dimerization [11]. The dimerized kinase domains can be a homodimer, which pairs with another EGFR, or a heterodimer, which pairs with other proteins of the ErbB family, such as ERBB2, ERBB3, and ERBB4. This dimerization event leads to trans-autophosphorylation of the kinase domain, catalyzing the transfer of a γ-phosphate from adenosine triphosphate (ATP) to conserved tyrosine residues at the C-terminal of EGFR. This phosphorylation event triggers a cascade of downstream signaling events, leading to the activation of various signaling pathways, ultimately influencing cell survival, proliferation, and differentiation [12,13].

**Fig. 1.**
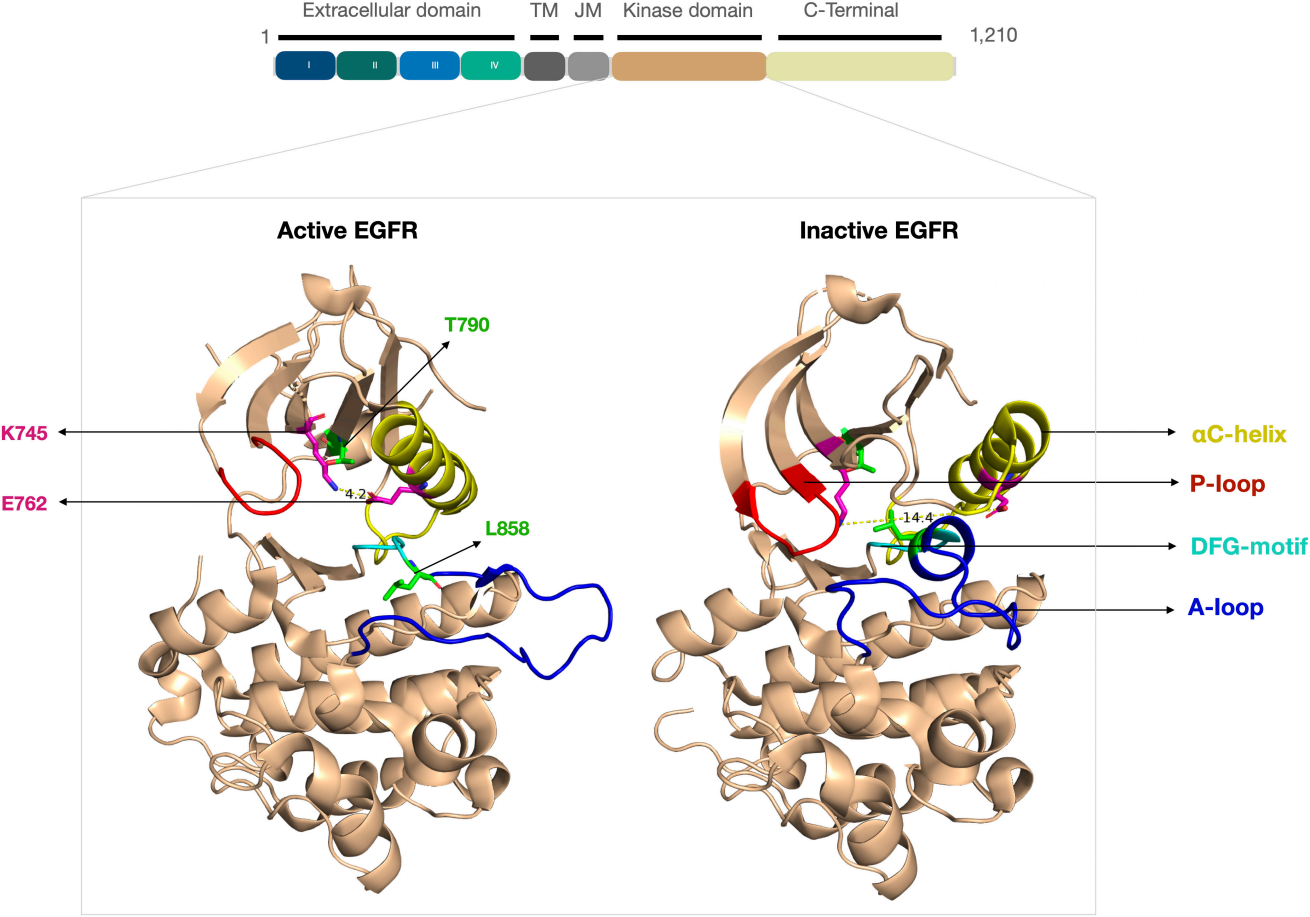
The epidermal growth factor receptor (EGFR) kinase sequence with various domains (extracellular region, transmembrane [TM] helix, juxtamembrane [JM] segment, kinase domain, and C-terminal tail), along with the crystal structure of active (Protein Data Bank [PDB] ID: 2GS2) and inactive (PDB ID: 4HJO) EGFR kinase domains, with various kinase substructures such as αC-helix, phosphate-binding loop (P-loop), Asp-Phe-Gly (DFG)-motif, and activation loop (A-loop), which are shown in yellow, red, cyan, and blue colors, respectively. Driver/resistance mutation sites and K745–E762 salt-bridge-forming residue positions are shown in green and magenta color, respectively.

Under physiological conditions, EGFR exists in a predominantly dimeric autoinhibited state and becomes activated upon the binding of a growth factor. These conformational transitions are governed by key structural motifs, including the phosphate-binding loop (P-loop), activation loop (A-loop), Asp-Phe-Gly (DFG)-motif, and αC-helix. The active conformation is characterized by the inward movement (“in”) of the αC-helix, an extended A-loop, and the formation of a critical salt bridge between K745 and E762, all of which stabilize ATP binding within the catalytic ATP-binding site. In contrast, the inactive conformation is marked by the outward movement (“out”) of the αC-helix, a collapsed A-loop, and a large increase in the distance between the K745 and E762 residues to 14.4 Å [14,15] (Fig. 1).

Crystallographic studies have substantially improved our understanding of structural transitions between active and inactive states of EGFR, as well as the potential intermediate conformations that exist between these states [16–19]. However, the static nature of crystallographic data has limitations in capturing the full extent of EGFR’s conformational dynamics. To address this, all-atom molecular dynamics (MD) simulations have been extensively employed to study the structural fluctuations and transitions of the EGFR kinase domain [14,20,21]. For instance, a study by Songtawee et al. [21] analyzed the asymmetric movement of EGFR dimers in both active and inactive states, revealing a decrease in structural fluctuations in the active dimer, whereas no notable conformational changes were observed in the inactive dimer. Another study, utilizing ~50 μs of MD simulations, examined the active-to-inactive transitions of EGFR, identifying intermediate conformations that correlated with hydrogen–deuterium (H/D) exchange data, a technique used to investigate protein folding and structural dynamics [14]. MD simulations, in particular, allow for the detailed analysis of protein motions and interactions at the atomic level. However, the accuracy of MD simulations relies on careful selection of parameters, such as the nonbonded cutoff distance, which determines the range of interatomic interactions considered. An appropriate cutoff distance is important for accurately representing interatomic interactions, including van der Waals forces and the real-space component of electrostatics, such as those involved in the K745–E762 salt bridge, which can substantially influence protein dynamics and function. Despite these advances, only a limited number of studies have leveraged microsecond- to millisecond-scale MD simulations to explore large-scale EGFR kinase domain folding and conformational transitions.

The abnormal structural transitions and stabilization of EGFR can also result from somatic mutations at critical residues. For example, L858, located within the A-loop, plays a crucial role in stabilizing the inactive EGFR conformation through hydrophobic interactions with N-lobe residues. Due to its functional importance, L858 is frequently mutated in patients with NSCLC, leading to constitutive kinase activation [5]. Other commonly observed mutations include exon 19 deletions, kinase domain duplications, and rare mutations such as G719X, L861Q, and S768I [22,23]. Various small-molecule kinase inhibitors have been designed to inhibit EGFR activation by targeting the ATP-binding site. However, prolonged use of these inhibitors frequently leads to the emergence of resistance mutations, the most notable being T790M. This secondary mutation in EGFR^L858R/T790M^ confers resistance to first- and second-generation ATP-competitive inhibitors that target the EGFR^L858R^ [24,25].

To circumvent ATP-binding-site resistance, non-ATP-competitive allosteric inhibitors have been developed. These inhibitors bind to an allosteric site distinct from the ATP-binding pocket, inducing conformational changes that render the kinase domain inactive. One such inhibitor, EAI001, selectively targets the EGFR^L858R/T790M^ mutant, exhibiting an half-maximal inhibitory concentration (IC50) value of 24 nM at 1-mM ATP concentration [18]. The aminothiazole moiety of EAI001 interacts directly with the T790M mutation, which is hypothesized to contribute to its selectivity toward EGFR^L858R/T790M^ over wild-type EGFR. A derivative of EAI001, EAI045, was subsequently developed with improved binding affinity, although it lacks selectivity. Additionally, other allosteric inhibitors such as JBJ-04-125-02 and JBJ-07-149 have demonstrated promising efficacy, exhibiting high binding affinities to EGFR^L858R/T790M^ with IC50 values of 0.26 nM and 1.1 nM, respectively [26,27]. Considering the importance of allosteric inhibitors, it is crucial to screen and develop new allosteric inhibitors with high binding affinity toward mutant EGFR^L858R/T790M^, which can change the conformation of EGFR as well. Given the therapeutic significance of allosteric inhibitors, ongoing research is essential to identify novel compounds with enhanced binding affinity for EGFR^L858R/T790M^. Additionally, the ability of these inhibitors to alter EGFR’s structural dynamics and favor its inactive conformation remains a critical area of investigation. Understanding these mechanistic insights will aid in the rational design of next-generation allosteric inhibitors that can effectively target drug-resistant EGFR mutants while minimizing off-target effects.

In this in silico study, we used all-atom (MD) simulations to accurately capture long-range interactions crucial for EGFR dynamics. We investigated the structural alterations in the inactive EGFR kinase domain caused by the L858R/T790M mutation and the impact of the allosteric inhibitor EAI001. MD simulations for 2 μs on the inactive-state structures of apo-EGFR^Wild^, apo-EGFR^L858R/T790M^, and EAI001-bound inactive EGFR^L858R/T790M^ revealed notable movements in critical substructures, including the αC-helix, A-loop, and P-loop. We analyzed the conformational landscape of EGFR^L858R/T790M^, its propensity for shifting toward active-like structural features, and the effect of the allosteric inhibitor EAI001 on modulating the conformational preferences of the inactive kinase domain. Finally, for the identification of potential allosteric inhibitors of EGFR^L858R/T790M^, we have standardized a virtual screening protocol involving the virtual screening of an allosteric kinase inhibitor library through docking, reranking their binding affinity with the scoring function (structure-guided machine-learning-based protein–ligand affinity predictor, SG-ML-PLAP) [28], and evaluation of the top candidates using MD simulations to analyze the structural impact of inhibitors on EGFR^L858R/T790M^ and the calculation of the Molecular Mechanics Generalized Born Surface Area (MM/GBSA) binding free energy (Δ*G*_bind_) to assess their potential as novel therapeutic agents.

## Materials and Methods

### Preparation of EGFR kinase structures for simulation

The inactive-state crystal structure (Protein Data Bank [PDB] ID: 5D41) of EGFR kinase with T790M mutation and bound to the allosteric inhibitor EAI001 was retrieved from the PDB [18]. The L858R mutation was modeled in the PDB structure 5D41 to prepare a double-mutant inactive EGFR with a driver mutation L858R and a resistance mutation T790M. To generate a wild-type inactive EGFR model, the Met790 mutation was reverted to its original residue Thr790. All the mutations were created with the help of Chimera software [29]. As the A-loop region of the 5D41 crystal structure was not resolved, the loop region was modeled through the built-in MODELLER extension in Chimera software. In the case of the active structure of EGFR kinase (PDB ID: 2GS2), 2 mutations L858R and T790M were modeled to create the apo-active EGFR^L858R/T790M^ system. All the structures were modeled for the missing residues with the help of the MODELLER tool [30] integrated in Chimera. Final systems for MD simulations included active apo-EGFR^Wild^, active apo-EGFR^L858R/T790M^, inactive apo-EGFR^Wild^, inactive apo-EGFR^L858R/T790M^, and inactive EAI001–EGFR^L858R/T790M^. The PDB structures, mutations, and simulation time used in this study are provided in Table 1.

**Table 1.** Information about the preparation of simulation systems, along with the simulation time

Simulation systems	Protein Data Bank ID	Mutations(s) modeled	Simulation time (μs)
Apo-active EGFR^Wild^	2GS2		2
Apo-active EGFR^L858R/T790M^	2GS2	L858R/T790M	2
Apo-inactive EGFR^Wild a^	5D41	M790T	2 (extended to 10)
Apo-inactive EGFR^L858R/T790M a^	5D41	L858R	2 (extended to 10)
EAI001-inactive EGFR^L858R/T790M a^	5D41	L858R	2 (extended to 10)

^a^ Each of the 3 inactive systems was simulated in 2 independent 1-μs runs for reproducibility.

### Preparation of ligand structures for simulation

The allosteric inhibitor EAI001 and other top kinase allosteric modulators selected through virtual screening in this study were prepared by adding hydrogens and Austin Model 1-Bond Charge Correction charges [31]. Ligand parameters were generated using the General Amber Force Field via the Antechamber module [32,33].

### MD simulations

MD simulation for all 5 systems (Table 1) was carried out using the AMBER 20 [34] package and ff14SB force field [35]. In each simulation, the structures were solvated with explicit TIP3P water molecules using an octahedral water box that extended 10 Å from the outermost protein atoms in all directions. The solvated system was neutralized by adding Na^+^ counter ions and additional Na^+^/Cl^−^ were added to the box to achieve 150 mM salt concentration. The solvated proteins were minimized for 5,000 cycles with the steepest descent and conjugate gradient. The restraint of 25 kcal mol^−1^ Å^−2^ was applied in the start of minimization and was reduced to 0 kcal mol^−1^ Å^−2^. After that, the system was heated gradually to achieve the temperature of 300 K over 100 ps of MD run in the NVT ensemble. During the heating process, the temperature was increased from 0 to 300 K with the restraint of 10 kcal mol^−1^ Å^−2^. The system was equilibrated in the NPT ensemble for 900 ps of MD run with restraint gradually decreasing from 25 to 0.1 kcal mol^−1^ Å^−2^. After this, a final 5-ns equilibration was done without restraints before starting the production step. A production dynamic was carried out without restraint for 2 μs. Electrostatic interactions were calculated using the Particle Mesh Ewald method, with a 16-Å nonbonded cutoff applied to real-space electrostatic and Lennard–Jones interactions; long-range electrostatics were treated by the Particle Mesh Ewald throughout. Because the K745–E762 salt-bridge distance samples have separations up to ~18 Å throughout the trajectories frequently exceeding standard short cutoff values, we performed a cutoff-sensitivity analysis for 700 ns (8, 13, and 16 Å) and retained 16 Å as a conservative, system-specific choice to avoid truncating this interaction within its frequently sampled distance range (Table S1). A 2-fs time step was used, with the SHAKE algorithm applied to bonds involving hydrogen atoms, temperature was controlled using a Langevin thermostat with a collision frequency of 2 ps^−1^, and pressure was maintained at 1 atm using a Monte Carlo barostat. Protonation states were assigned at pH 7.0 using the LEaP module of AMBER 20. To assess reproducibility, replicate simulations (1 μs each, independently randomized initial velocities) were performed for the 3 main simulation systems.

### Analysis of MD simulation trajectories

Analysis of MD trajectories was done in terms of (a) root mean square deviation (RMSD) of backbone Cα-atoms, which measures the distance of atoms throughout the simulation with relation to the reference structure to analyze the overall stability and (b) root mean square fluctuation (RMSF) of Cα-atoms, which measures the overall displacement of atoms throughout the simulation with relation to the reference structure to analyze flexible regions of protein structure. (c) The distance between salt-bridge-forming residues K745(NZ) and E762(CD) was also calculated throughout the 2-μs production steps. (d) Residue contact frequency and hydrogen bond occupancy were calculated for all 10 selected candidate compounds and EAI001.

### Principal component analysis

Principal component analysis (PCA) was performed to analyze dominant protein motion patterns during the MD simulation production phase. PCA reduces the system’s dimensionality and identifies functionally relevant conformational changes. Analysis was conducted using cpptraj, and results were visualized using Matplotlib in Python [34,36,37].

### Two-dimensional free energy surface analysis

Two-dimensional (2D) free energy surfaces (FESs) were constructed to characterize the conformational landscapes of the 3 simulation systems: apo-inactive EGFR^Wild^, apo-inactive EGFR^L858R/T790M^, and EAI001-bound EGFR^L858R/T790M^. The K745–E762 salt-bridge distance and the E762–D855 inter-residue distance were used as collective variables (CVs). The E762–D855 distance was chosen as a directional reporter of αC-helix positioning. Free energy values were computed from the normalized probability distributions of the CVs extracted from the 2-μs production trajectories using the relation *G* = −*k*_B_ *T*ln*P*, where *k*_B_ is the Boltzmann constant, *T* is the simulation temperature, and *P* is the probability of each bin. Conformational states were classified based on joint thresholds applied to both collective variables: Frames were assigned as inactive-like (K745–E762 > 13 Å and E762–D855 > 12 Å), active-like (K745–E762 < 7 Å and E762–D855 < 7 Å), or intermediate-like (all remaining frames), based on structural benchmarks from inactive (K745–E762 ~14.4 Å) and active (K745–E762 ~4 Å) EGFR crystal structures. The fraction of frames in each conformational state was quantified for all 3 systems. Confidence intervals were estimated using the normal approximation to the binomial distribution.

### MM/GBSA for Δ*G*_bind_ calculations

The MM/GBSA method was used to estimate the relative Δ*G*_bind_ of EAI001 and selected allosteric modulators with EGFR^L858R/T790M^ (100 ns) [38] using the module of AMBER 20 software. Trajectory frames were saved every 10 ps (0.01 ns per frame), yielding 10,000 frames over the 100-ns production run; for MM/GBSA analysis, every fifth frame was extracted, resulting in 2,000 snapshots spanning the full 100-ns trajectory. The Δ*G*_bind_ was calculated using the following equation:

$$\Delta G_{\text{bind}}=\Delta G_{\text{complex}}-\left(\Delta G_{\text{protein}}+\Delta G_{\text{ligand}}\right)$$ (1)

This equation is for calculating the Δ*G*_bind_ difference between 2 states, that is, bound state and free state of ligand and protein. Here, Δ*G*_bind_ represents the relative Δ*G*_bind_ of the protein–ligand complex, Δ*G*_complex_ is free energy of the bound complex, Δ*G*_protein_ is free energy of unbound protein, and Δ*G*_ligand_ is free energy of unbound ligand. Generalized Born solvation was modeled using igb=2 with a solute dielectric constant of 1, solvent dielectric constant of 80, and a salt concentration of 0.150 M. Entropic contributions were not included, and the resulting MM/GBSA Δ*G*_bind_ are therefore interpreted as approximate, protocol-dependent values suitable for relative ranking of compounds rather than absolute binding affinities.

### Virtual screening using docking simulations

#### Preparation of receptor

The minimized structure of the EAI001-bound inactive EGFR^L858R/T790M^ complex was used for docking studies. The EAI001 inhibitor was removed from the binding pocket to prepare the receptor for docking new ligands. The receptor was prepared for docking using AutoDock Tools [39], which involves adding hydrogen atoms, assigning charges, and converting the structure into a format compatible with the docking software.

#### Preparation of the chemical compound library

To screen allosteric modulators for inactive EGFR^L858R/T790M^, ChemDiv’s (https://www.chemdiv.com/) library of 26k allosteric kinase inhibitors was used for virtual screening. Each compound from the ChemDiv library was assigned an internal sequential identifier, and the top-ranked hits are referred to using these codes throughout the manuscript. All the compounds were minimized using openbabel before preparing them for docking [40]. The ligands were prepared for docking using the prepare_ligand.py script from the ADFR suite [41], which converts the ligand structures into the appropriate format for AutoDock Vina.

#### Docking

For docking, a grid was generated, which was kept ligand centric; here, the ligand was allosteric ligand, i.e., EAI001, having coordinates surrounded by allosteric pocket. The re-docking of mutant EGFR^L858R/T790M^–EAI001 complex was done for the validation of docking parameters. Finally, the grid size was kept at 20 × 20 × 20 Å and the exhaustiveness was set to a default setting of 10. The docking was performed by AutoDock Vina, a maximum of 9 ligand poses were generated, and the top-scoring pose was considered for further analysis [42].

#### SG-ML-PLAP re-scoring

The docked compounds were re-scored using SG-ML-PLAP, a machine-learning-based scoring function developed in our earlier work [28], which uses extended connectivity interaction fingerprints derived from protein–ligand structural complexes as input features. The model was trained and validated on the PDBbind dataset, and outputs predicted binding-affinity values correlated with p*K*_d_/p*K*_i_/IC50. The reported scores are unitless values on the model’s regression scale and are not equivalent to kilocalories per mole; they serve as a relative ranking metric to prioritize compounds with higher predicted binding affinity than EAI001. SG-ML-PLAP has been benchmarked on the comparative assessment of scoring functions dataset, demonstrating superior performance compared to conventional docking scoring functions. It should be noted that the model was trained on the broader PDBbind dataset and was not specifically calibrated for EGFR allosteric ligands, hence the scores reported here should therefore be interpreted as relative rankings rather than absolute binding-affinity values.

## Results

### Dynamics of the wild-type EGFR starting from the active and inactive states

In order to understand the conformational dynamics of the EGFR, we carried out a 2-μs MD simulation on the wild-type EGFR starting from its active-state crystal structure and modeled conformation for the inactive state. The model for the wild-type inactive structure of EGFR was built using the crystal structure of inhibitor-bound EGFR^T790M^ as a template. We calculated the RMSD of the protein backbone to analyze the stability of the simulations. For both the active and inactive wild-type EGFR, the RMSD exhibited higher fluctuations up to 1 μs of the simulation time and stabilization was observed at the later stages of the simulations (Fig. S1A). The analysis of local flexibility of EGFR substructural regions using RMSF showed high fluctuations in the αC-helix region of the active EGFR structure, and on the other hand, the P-loop and A-loop regions displayed high fluctuations in the simulation of inactive EGFR, suggesting their role in the EGFR conformational state transition (Fig. S1B).

**Fig. 2.**
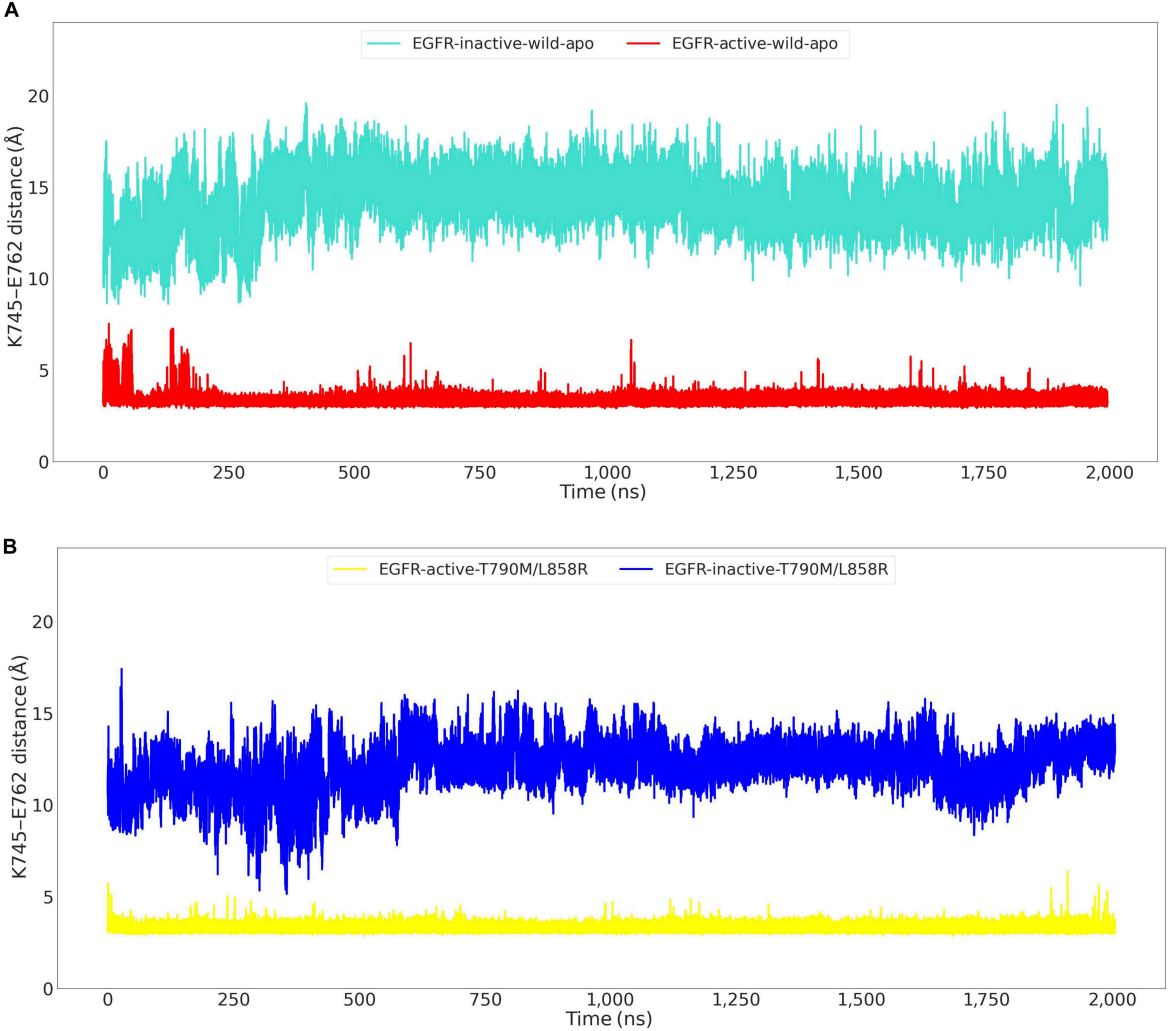
Time series of the distance between the NZ atom of K745 and the CD atom of E762 (salt-bridge-forming residues) in the EGFR kinase domain. (A) Active and inactive apo-EGFR^Wild^ over 2-μs simulations. (B) Active and inactive apo-EGFR^L858R/T790M^ over 2-μs simulations.

The critical structural feature that distinguishes the active from the inactive conformational state of EGFR is the formation of a salt bridge between the residues K745 and E762 within the kinase domain. Hence, we analyzed the distance between these salt-bridge-forming residues throughout the 2-μs simulation for both active and inactive EGFR structures. When starting from the active state, while the average salt-bridge distance was 3.39 Å, indicating its general formation, we observed its frequent fluctuations. Notably, within the initial 250 ns, the salt-bridge distance transiently extended up to 7.5 Å, suggesting periods of instability. This dynamic behavior was observed throughout the entire 2-μs simulation, with the salt bridge fluctuating from its stabilized states to exhibit frequent occurrences of conformational states where the K745–E762 distance extended to approximately ~6.5 Å (Fig. 2A). In contrast, simulations initiated from the inactive wild-type EGFR structure displayed a large distance between salt-bridge-forming residues, with an average of 14.27 Å (Fig. 2A), reflecting the absence of a salt bridge. The time-series plots of the K745–E762 distance revealed the fluctuation distance from 3 to 6.5 Å in a few places in the active state, indicating formation and breakage of the salt bridge, while the salt bridge was completely absent in the simulations, which started from the inactive state. Complementary to these findings, and further illustrating the distinct conformational characteristics of the active/inactive state, analysis of the K745–E762 distance with a 13-Å threshold (Fig. S2, 1-μs simulation) showed that the active wild-type EGFR maintained the salt-bridge-forming residue distance predominantly around 3 to 5 Å, extending up to ~6 Å, whereas the inactive wild-type EGFR structure consistently displayed a distance of approximately 11 Å.

### Dynamics of the double-mutant EGFR^L858R/T790M^ starting from the active and inactive states

Following the characterization of wild-type EGFR dynamics, we next investigated the L858R/T790M double mutant, which combines a common driver mutation (L858R) with a known resistance mutation (T790M). The overall stability of the mutant systems, analyzed through RMSD, showed relatively greater stabilization compared to their wild-type counterparts (Fig. S3A). This suggests that the presence of these mutations contributes to a relatively stable conformational state. Analysis of the fluctuations in the local regions of EGFR further differentiated the mutant EGFR dynamics from wild type. Notably, in the case of mutant EGFR in the active state, we observed higher fluctuations across substructural regions, including αC-helix, A-loop, and at the latter part of the P-loop, as compared to inactive mutant EGFR (Fig. S3B).

Analysis of the distance between K745 and E762 revealed the presence of a highly stable salt bridge in the active state of the L858R/T790M double mutant, while the corresponding salt bridge was absent in the inactive state (Fig. 2B). For the active double-mutant EGFR, an average distance of 3.31 Å was observed across the 2-μs simulation. Notably, unlike the frequent occurrences of conformations with K745–E762 distances extending up to 6.5 Å seen in the active state, the mutant almost entirely eliminated such conformations, underscoring a markedly more constrained and persistently formed salt bridge. Conversely, for the inactive double-mutant EGFR, the time-series plot of the distance between salt-bridge-forming residues across the 2-μs simulation showed its stabilization around ~12 Å, with an average distance of 12.08 Å (Fig. 2B). While this still indicates an inactive-like state, analysis of the K745–E762 distance over the 2-μs trajectory revealed that the distance had reduced to ~6 Å at several time points, and the shift toward a slightly shorter average distance compared to the wild-type inactive EGFR (average 14.27 Å) indicated a conformational shift toward the active-like state. These results indicate that the double mutant is not only more stable in the active state compared to the wild type but, unlike wild-type EGFR, it also has a propensity for conformational shift toward the intermediate active-like state even when simulations were started from the inactive state. Whether a complete inactive-to-active transition would be observed at longer time scales remains an open question beyond the scope of the current simulations. These results provide a theoretical rationale for the pathogenic aberrant phosphorylation by the EGFR^L858R/T790M^ in NSCLC. Further analysis of the K745–E762 salt-bridge distance with a 13-Å threshold (Fig. S4, 1-μs simulation) showed that the active L858R/T790M mutant structure showed sharp stabilization at an average distance of ~3 Å, while the inactive mutant structure displayed a highly dynamic and largely open conformation, qualitatively similar to wild-type inactive EGFR (Fig. S2).

### Dynamics of EGFR^L858R/T790M^ bound to allosteric inhibitor EAI001

**Fig. 3.**
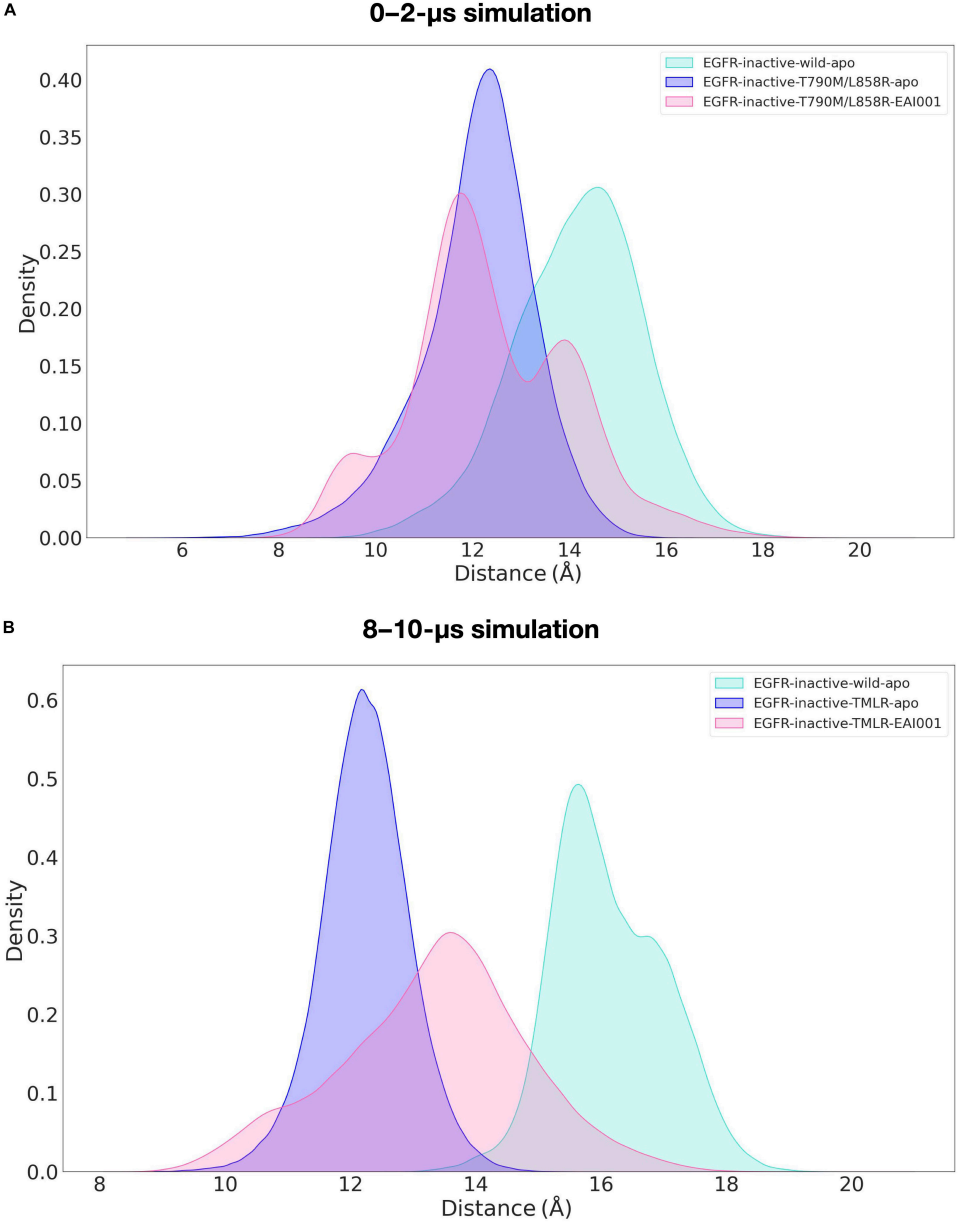
Kernel density distributions of the K745–E762 salt-bridge distance in inactive EGFR simulations. The distance between the NZ atom of K745 and the CD atom of E762 was calculated for conformations sampled during (A) 0- to 2-μs and (B) 8- to 10-μs simulations of apo-inactive EGFR^Wild^ (cyan), apo-inactive EGFR^L858R/T790M^ (blue), and EAI001-bound EGFR^L858R/T790M^ (pink). The plots illustrate how the mutant and inhibitor-bound systems differ in their preferred K745–E762 separations over early and late simulation windows.

Since the double mutant has high propensity for aberrant phosphorylation and is resistant to orthosteric inhibitors that bind at the ATP-binding site of the kinase, allosteric inhibitors like EAI001 have been developed. Allosteric inhibitors, which act by stabilizing an inactive enzyme conformation, represent a critical alternative therapeutic approach. Hence, we wanted to analyze the molecular basis of allosteric inhibition by carrying out a 2-μs MD simulation on the EAI001-bound inactive-state structure of EGFR^L858R/T790M^ double mutant. The K745–E762 salt bridge is crucial for stabilizing the active conformation of the EGFR kinase domain and facilitating ATP binding. Hence, the distance between these 2 salt-bridge-forming residues was used as reaction coordinate to monitor conformational shifts between inactive-like and intermediate/active-like substates in the EAI001-bound double mutant. Figure 3A shows the overlapped density plots for the distribution of K745–E762 distances observed for the inactive apo-EGFR^Wild^, inactive apo-EGFR^L858R/T790M^, and inactive EAI001–EGFR^L858R/T790M^. As explained earlier, in wild type, the average distance was 14.27 Å and minimum of 10 Å, indicating a stable inactive conformation. In contrast, the apo-EGFR^L858R/T790M^ mutant showed a decreased average distance of ~12 Å and minimum of 7.5 Å, suggesting that the mutations promote a shift toward an active-like conformation. However, the EAI001-bound EGFR^L858R/T790M^ exhibited a more complex behavior, with 3 distinct peaks in the salt-bridge distance distribution: a small peak at ~9.5 Å, a large peak at ~12 Å, and another peak at ~14 Å. This suggests that the inhibitor modulates the dynamics of the mutant EGFR kinase, inducing a conformational shift toward the inactive state (Fig. 3A). Furthermore, extending the simulation to 10 μs and analyzing the last 2-μs time frame (8 to 10 μs) (Fig. 3B) revealed a more pronounced peak around ~14 Å for the EAI001-bound EGFR^L858R/T790M^, indicating that over longer time scales, the inhibitor stabilizes inactive conformation. Consistent with this, the fraction of frames in the inactive population EAI001-bound EGFR^L858R/T790M^ (K745–E762 > 13 Å) increases from 33.6% in the 0- to 2-μs window to 61.9% in the 8- to 10-μs window, while the intermediate population (7 to 13 Å) decreases from 66.4% to 38.1%, with no active-like frames (<7 Å) observed in either window (Table S2).

To collectively characterize the conformational landscapes across all 3 systems, we performed 2D-FES analysis of 2-μs trajectories, computed using the K745–E762 salt-bridge distance and the E762–D855 inter-residue distance as CVs. The E762–D855 distance serves as a directional reporter of αC-helix positioning; shorter values (~7 to 10 Å) indicate an inward, activation-competent αC-helix, while longer values (>12 Å) indicate an outward, inactive-like αC-helix. Together, these 2 CVs reveal distinct conformational preferences across the 3 systems (Fig. S5). Conformational states were defined using both CVs: Frames with K745–E762 > 13 Å and E762–D855 > 12 Å were classified as inactive-like, those with K745–E762 < 7 Å and E762–D855 < 7 Å as active-like, and all remaining frames as intermediate-like (Methods).

In the apo wild-type inactive system, 80.5 ± 0.2% of frames are classified as inactive-like, with the remaining 19.5 ± 0.2% being intermediate-like, and no active-like frames were observed (Table S3 and Fig. S5A). This confirms that the wild-type structure faithfully maintains the inactive conformation over the simulation time scale and serves as a positive control. In the apo-EGFR^L858R/T790M^ mutant, 99.9 ± 0.0% of frames are classified as intermediate-like, with only 0.1% retaining inactive-like geometry (Table S3 and Fig. S5B). This indicates that the double mutation almost completely destabilizes the inactive-like basin, driving the kinase into conformations with a more inward αC-helix position even in the absence of ligand, consistent with the constitutively activating character of EGFR^L858R/T790M^. When EAI001 is bound, the free energy landscape shifts back toward longer K745–E762 and E762–D855 values, with the basin elongated along the K745–E762 axis (~8 to 18 Å) reflecting broader conformational heterogeneity. The inactive-like population recovers to 15.6 ± 0.2% and the intermediate-like population constitutes 84.4 ± 0.2% of frames, with no active-like frames observed in either the apo mutant or EAI001-bound system (Table S3 and Fig. S5C). Although the inactive-like fraction in the EAI001-bound system remains lower than in the wild type, it represents a substantial recovery relative to the apo mutant (0.1% to 15.6%), indicating that EAI001 partially restores the inactive-like basin and allosterically stabilizes a less activation-prone αC-helix conformation of EGFR^L858R/T790M^. Note that the 2D inactive-like criterion applied here is more conservative than the one-dimensional K745–E762-only criterion used in the time-windowed analysis earlier, as it requires simultaneous satisfaction of both distance thresholds and is done on initial 2-μs trajectories; the 2 analyses are therefore complementary rather than directly comparable. Taken together, these FES results demonstrate a pronounced shift in conformational preference away from the inactive-like basin in EGFR^L858R/T790M^ and a partial yet statistically significant reversal of this shift upon EAI001 binding within the simulated time scales.

**Fig. 4.**
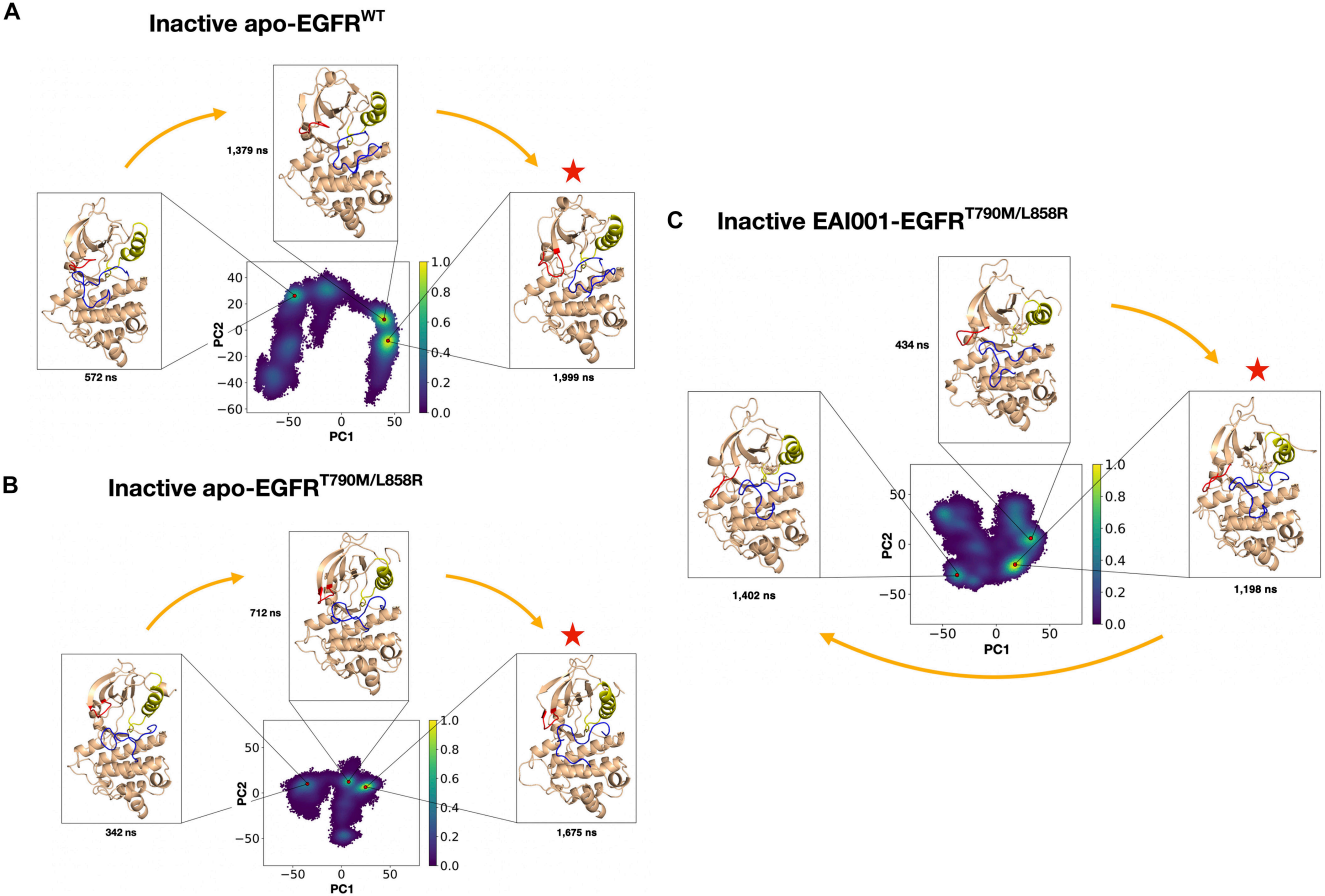
Principal component analysis (PCA) of EGFR kinase domain dynamics. The conformational landscapes are projected onto the first 2 principal components (PC1 and PC2), with the color scale indicating the normalized probability density of sampled conformations. The red star marks a representative structure extracted from a highly populated region for (A) inactive apo-EGFR^Wild^, (B) inactive apo-EGFR^L858R/T790M^, and (C) inactive EAI001-bound EGFR^L858R/T790M^; the corresponding conformations are shown around each plot.

Next, to analyze various substructural dynamics of the kinase domain, we employed PCA to identify the major collective motions of the protein during the simulations. Figure 4 shows the PCA plot, where the yellow regions in the plot indicate clusters of similar conformations sampled during the simulations. We extracted representative structures from the highly populated clusters in each simulation system (wild type, apo mutant, and inhibitor-bound mutant) to compare the conformations of key substructures (P-loop, A-loop, DFG-motif, and αC-helix) across these representative structures and gain insights into how the L858R/T790M mutation and the allosteric inhibitor influence the conformational dynamics of the EGFR kinase domain.

**Fig. 5.**
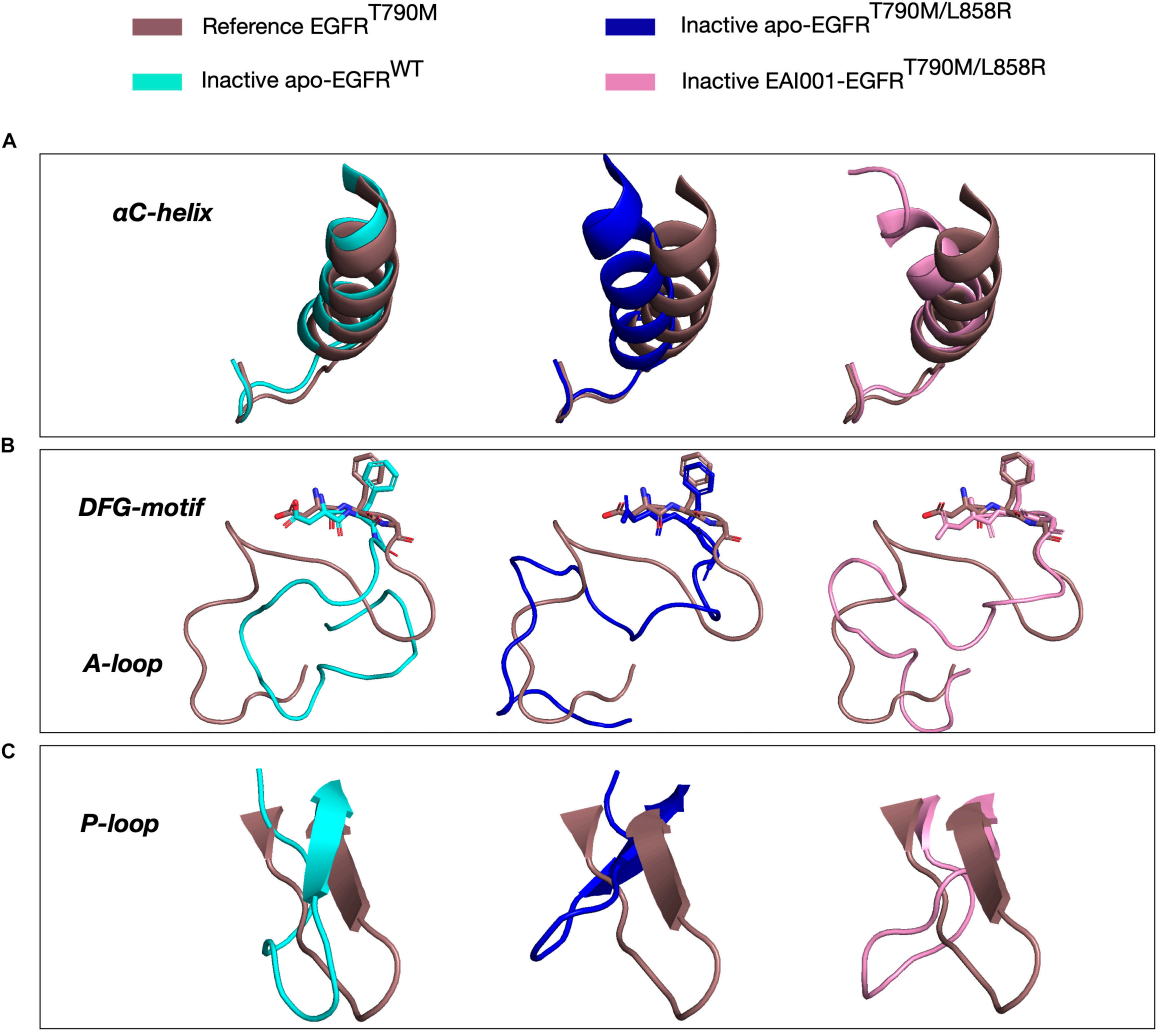
Superimposition of the EGFR kinase conformations showing αC-helix (A), DFG-motif and A-loop (B), and P-loop (C) (taken from the highly populated PCA cluster) onto the inactive EGFR crystal structure (PDB ID: 5D41) taken as a reference.

Analysis of the αC-helix, a key structural element involved in kinase activation, revealed distinct movements across different systems. In the inactive apo-EGFR^Wild^, the 3 major conformations obtained from the PCA clusters at 572 ns, 1,379 ns, and 1,999 ns showed no notable movement of the αC-helix (Fig. 4A). This observation was further supported by the overlap of the conformation from the highest PCA cluster with the reference crystal structure 5D41 with modeled A-loop (Fig. 5A). Interestingly, in the inactive apo-EGFR^L858R/T790M^, a substantial inward movement of the αC-helix was observed as the simulation progressed, suggesting a shift toward an active-like conformation (Fig. 4B). This inward movement was also evident in the structural overlap with the reference crystal structure (Fig. 5A). In contrast, for the EAI001-bound inactive EGFR^L858R/T790M^, a substantial loss of the starting αC-helix was observed in the majority of the population, as seen in the structure at 1,198 ns (Figs. 4C and 5A). However, a small cluster formed at the later stage of the simulation exhibited reformation of the αC-helix (Fig. 4C).

To further analyze the changes in the secondary structure of the αC-helix, we calculated the α-helix occupancy of the inactive EAI001–EGFR^L858R/T790M^ throughout the simulation. The results (Fig. S6) clearly showed the decrease in the α-helix occupancy at the start of the αC-helix upon inhibitor binding, suggesting the formation of a local disordered state in the mutant EGFR [14,43]. This observation is consistent with previously reported inactive EGFR structures with shorter αC-helices (PDB ID: 2GS7, 4HJO).

The A-loop, another crucial regulatory element, was modeled in our simulations due to missing coordinates in the inactive crystal structure. The MD simulations provided valuable insights into its flexibility. Our analysis revealed high flexibility of the modeled A-loop in all 3 systems. In the inactive apo-EGFR^Wild^, the A-loop contracted in the center (Figs. 4A and 5B), potentially representing an intermediate position before forming a short helix at its N-terminal and blocking the ATP-binding site. A similar, albeit less pronounced, contraction was observed in the EAI001-bound EGFR^L858R/T790M^ (Figs. 4C and 5B). In contrast, the A-loop of the apo-EGFR^L858R/T790M^ mutant was more relaxed and extended toward the left-hand side (Figs. 4B and 5B). A short α-helix formed at the N-terminal portion of the A-loop (Gly857 to Gly863) is a key element that prevents the EGFR kinase from being active until the ligand binds and induces the conformational change [43]. Since no α-helix formation was observed at the A-loop in any of the systems, we analyzed the changes in its secondary structure. While no helix formation was observed, an increase in turn-like conformation (prior to formation of helix) was observed in both the inactive apo-EGFR^Wild^ and the EAI001–EGFR^L858R/T790M^ compared to the apo-EGFR^L858R/T790M^, suggesting an increased propensity for helix formation (Fig. S7). This was confirmed by the formation of an α-helix at the end of an 8.5-μs simulation of the EAI001-bound EGFR^L858R/T790M^ (Fig. S8), which was not observed in the other 2 systems even after extending the simulations to 10 μs. No notable movement of the DFG-motif was observed in any of the simulation systems (Fig. 5B).

In the inactive EGFR state, the P-loop is folded inwards and interacts with the αC-helix through hydrophobic interactions, maintaining the αC-helix-out conformation [44]. Our simulations revealed that the P-loop in the wild-type inactive EGFR was slightly coiled and moved inwards relative to the apo-EGFR^L858R/T790M^ and EAI001–EGFR^L858R/T790M^. The P-loop in the EAI001-bound system was not folded inwards to the same extent, with its movement falling somewhere between the apo-EGFR^L858R/T790M^ and apo-EGFR^Wild^ (Fig. 5C). This suggests that both the mutation and the inhibitor can influence the conformation and dynamics of the P-loop, potentially affecting its interaction with the αC-helix and the overall stability of the inactive state.

Lastly, we also analyzed the distance between salt-bridge-forming residues in representative conformations extracted from the most populated PCA clusters (Fig. S9). The largest distance (14.4 Å) was observed in the wild type, followed by 13.9 Å in the EAI001-bound mutant. The apo mutant showed the shortest distance (11.2 Å), reinforcing the notion that the mutation favors a conformation closer to the active state. The intermediate distance in the EAI001-bound mutant, along with the multimodal distribution, suggests that the inhibitor induces a more dynamic behavior of the salt bridge, preventing it from adopting the active conformation.

### Convergence and statistical robustness of EGFR simulations

Independent replicate simulations (1 μs each) of the 3 main systems, apo-inactive EGFR^Wild^, apo-inactive EGFR^L858R/T790M^, and EAI001-bound EGFR^L858R/T790M^, show RMSD and K745–E762 distance time series that are very similar to the corresponding primary trajectories and do not reveal additional slow transitions, providing a basic reproducibility check for the simulation runs (Fig. S10A to F). To further quantify convergence of the K745–E762 distance, we performed block-averaging analyses for each 10-μs trajectory. Over the full 10 μs, the block-averaged K745–E762 distances are 14.44 ± 0.87 Å (apo-inactive EGFR^Wild^), 12.41 ± 0.57 Å (apo-inactive EGFR^L858R/T790M^), and 12.57 ± 1.22 Å (EAI001-bound EGFR^L858R/T790M^), indicating well-defined mean values within the sampled time. When the analysis is restricted to the last 2 μs (8 to 10 μs), the block-averaged distances become 14.70 ± 0.87 Å, 12.24 ± 0.39 Å, and 13.33 ± 1.25 Å, respectively, revealing a progressive shift of the EAI001-bound mutant toward larger, more inactive-like K745–E762 separations at longer times. Together, the replicate simulations and block-averaging statistics support the robustness of the reported K745–E762 distance differences within the simulated time scale; while extremely rare, slower transitions cannot be fully excluded.

### A-loop modeling and validation

The A-loop (residues ~855 to 877) was absent from the 5D41 crystal structure and was modeled using MODELLER integrated in Chimera. The top-scoring model was selected based on a normalized discrete optimized protein energy score of −1.46146, which indicates a reliable structural model. Ramachandran analysis of the 19 A-loop residues in the starting post-minimization structure revealed that 10/19 residues (52%) fall in favored regions (Fig. S11A). Four residues (K860, A864, E866, and K875) fall in the disallowed region of the Ramachandran plot. Their dihedral angles relax to favored and additionally allowed regions within the 100-ns simulation trajectory, as confirmed by analysis of 10,000 trajectory frames, in which 68% (129,662/190,000) of all A-loop backbone dihedral angle pairs fall in favored Ramachandran regions (Fig. S11B). The modeled loop is shown in orange overlaid with the original (resolved in apo-inactive EGFR) loop position in black in Fig. S12A; while not identical to the template conformation, the modeled loop adopts a broadly similar fold without deviating into an unphysical conformation. The post-100-ns position is shown in blue, confirming that the loop stabilizes in a conformation proximal to the inactive-state template region rather than drifting away from it throughout simulation (Fig. S12B).

### Virtual screening of allosteric kinase inhibitors with mutant EGFR^L858R/T790M^ kinase revealed top allosteric binders

The development of conventional tyrosine kinase inhibitors depends upon how strongly they bind to the kinase orthosteric pocket to carry out competitive inhibition against ATP. However, the EGFR allosteric inhibitors work by altering the conformation of mutant EGFR^L858R/T790M^ and stabilizing the inactive conformational state. Hence, for the development of better allosteric inhibitor/modulators, it is crucial to analyze its ability to prevent the shift of EGFR^L858R/T790M^ into an active-like state along with the high binding affinity to the allosteric pocket. Therefore, we developed a virtual screening protocol where high-affinity kinase allosteric binders are screened through molecular docking studies, followed by MD simulations to analyze their effect on the dynamics of EGFR^L858R/T790M^ to stabilize it in an inactive conformational state.

In order to identify novel allosteric modulators with potentially higher affinity for the EGFR^L858R/T790M^ mutant than the known inhibitor EAI001, we conducted a virtual screening. For that, we took the minimized conformation of inactive EGFR^L858R/T790M^, which was minimized with EAI001 to adjust the inhibitor in the pocket of L858R/T790M mutant EGFR. Before screening a library of compounds, we first validated our docking protocol by re-docking EAI001 into the allosteric pocket of EGFR^L858R/T790M^ using AutoDock Vina. The re-docked ligand pose closely resembled the original binding pose observed in the minimized structure (Fig. S13A), confirming the reliability of our docking parameters. The predicted Δ*G*_bind_ for EAI001 was −9.9 kcal/mol. Analysis of the re-docked complex revealed key interactions between EAI001 and the allosteric binding-pocket residues (Fig. S13B). These interactions include hydrogen bonds with Asp855 and Lys745 and hydrophobic interactions with Met790, Leu788, Met766, Leu777, Ile759, Glu762, and Ala763. Importantly, this interaction pattern is consistent with that observed in the minimized EAI001–EGFR^L858R/T790M^ complex and crystal structure of the EAI001–EGFR^T790M^ complex, further validating our docking approach. With a validated docking protocol, we proceeded to screen a library of allosteric kinase inhibitors to identify promising candidates that could bind to the allosteric site of the EGFR^L858R/T790M^ mutant with high affinity.

**Fig. 6.**
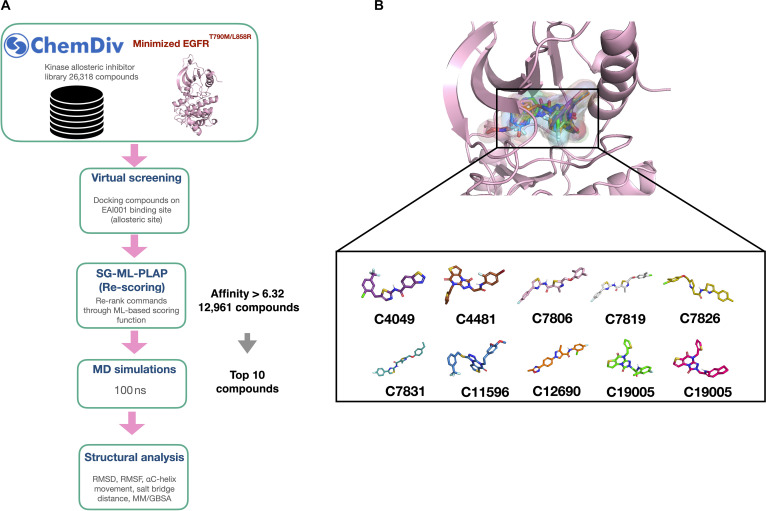
Virtual screening pipeline for finding the potential allosteric inhibitors for EGFR^L858R/T790M^ (A). RMSD, root mean square deviation; RMSF, root mean square fluctuation; MD, molecular dynamics. The top 10 compounds selected for simulations with the binding affinity (SG-ML-PLAP score) higher than EAI001 (B).

A library of 26,318 kinase allosteric inhibitors from ChemDiv were docked in the allosteric site of the EGFR^L858R/T790M^ kinase domain (Fig. 6A). Since the docking scores often do not show good correlation with the experimental binding affinities, we used an ML-based scoring function SG-ML-PLAP to refine our selection and improve the accuracy of binding-affinity predictions. In our earlier work, SG-ML-PLAP was trained and validated on a large dataset of protein–ligand complexes with known binding affinities [28]. It was found that, out of the 26,318 compounds, 12,961 had SG-ML-PLAP scores above that of EAI001. From this list of top-scoring compounds, we selected the top 10 compounds exhibiting higher predicted binding affinities than EAI001. These compounds showed binding energy scores ranging from 9.61 to 9.27, compared to 6.32 for EAI001, suggesting stronger interactions with the allosteric pocket. The AutoDock VINA and SG-ML-PLAP scores of these selected compounds are listed in Table 2. As intended, all 10 compounds were predicted to bind within the allosteric pocket of EGFR (Fig. 6B). To further assess the suitability of these compounds as potent allosteric modulators of EGFR^L858R/T790M^, we evaluated their drug-likeness and physiochemical properties. The radar plot (Fig. S14) generated by ADMETLAB3.0 for the predicted properties revealed that all 10 compounds fell within the acceptable range, indicating favorable drug-like characteristics [45]. This suggests that these compounds not only exhibit high affinity for the EGFR^L858R/T790M^ allosteric site but also possess promising drug-like properties, warranting further investigation as potential lead compounds for drug development.

**Table 2.** AutoDock Vina scores and structure-guided machine-learning-based protein–ligand affinity predictor (SG-ML-PLAP) predicted score for the binding affinity of EAI001 and top 10 compounds obtained after docking

Name	SG-ML-PLAP score	Docking score (kcal/mol)
C4481	9.61	−7.2
C19005	9.54	−6.4
C11596	9.34	−7.4
C7806	9.34	−6.5
C7826	9.34	−7.0
C7831	9.34	−6.1
C7819	9.32	−5.9
C12690	9.31	−4.7
C4049	9.30	−7.3
C19017	9.27	−6.7
EAI001	6.32	−9.9

To further evaluate the top 10 allosteric modulators identified through virtual screening, we performed 100-ns MD simulations for each compound in complex with the inactive EGFR^L858R/T790M^ kinase. This allowed us to assess the stability of the complexes and analyze the impact of the modulators on the EGFR^L858R/T790M^ kinase dynamics. Analysis of the RMSD of both the protein and the ligands revealed that most complexes were stable throughout the 100-ns simulations (Fig. S15A and B). All compounds, except for C7806 and C7826, showed average RMSD values within 2.5 Å, indicating stable binding. The EGFR^L858R/T790M^ kinase itself also remained stable in all simulations, with average RMSD values within 3 Å, except for C7806. Next, we analyzed the RMSF values of the EGFR^L858R/T790M^ residues simulated with potential allosteric modulators to understand the atomic fluctuation of substructural regions. C7806, C7831, C7826, and C4481 showed high fluctuation in αC-helix; C11596, C19005, C7826, and C12690 showed high fluctuation in A-loop; and C12960, C19017, and C19005 showed high fluctuation in P-loop (Fig. S15C). The superimposition of the final conformations of EGFR^L858R/T790M^ after 100-ns simulations with the initial minimized structure (Fig. S16) revealed substantial structural changes in αC-helix induced by some of the compounds. Notably, C7806, C4049, and C19005 caused a pronounced outward movement of the αC-helix, even greater than that observed with EAI001. Also, C7831 led to a reduction in the αC-helix. Overall, structural changes caused by allosteric modulators in the mutant EGFR^L858R/T790M^ were observed in the majority of cases.

**Fig. 7.**
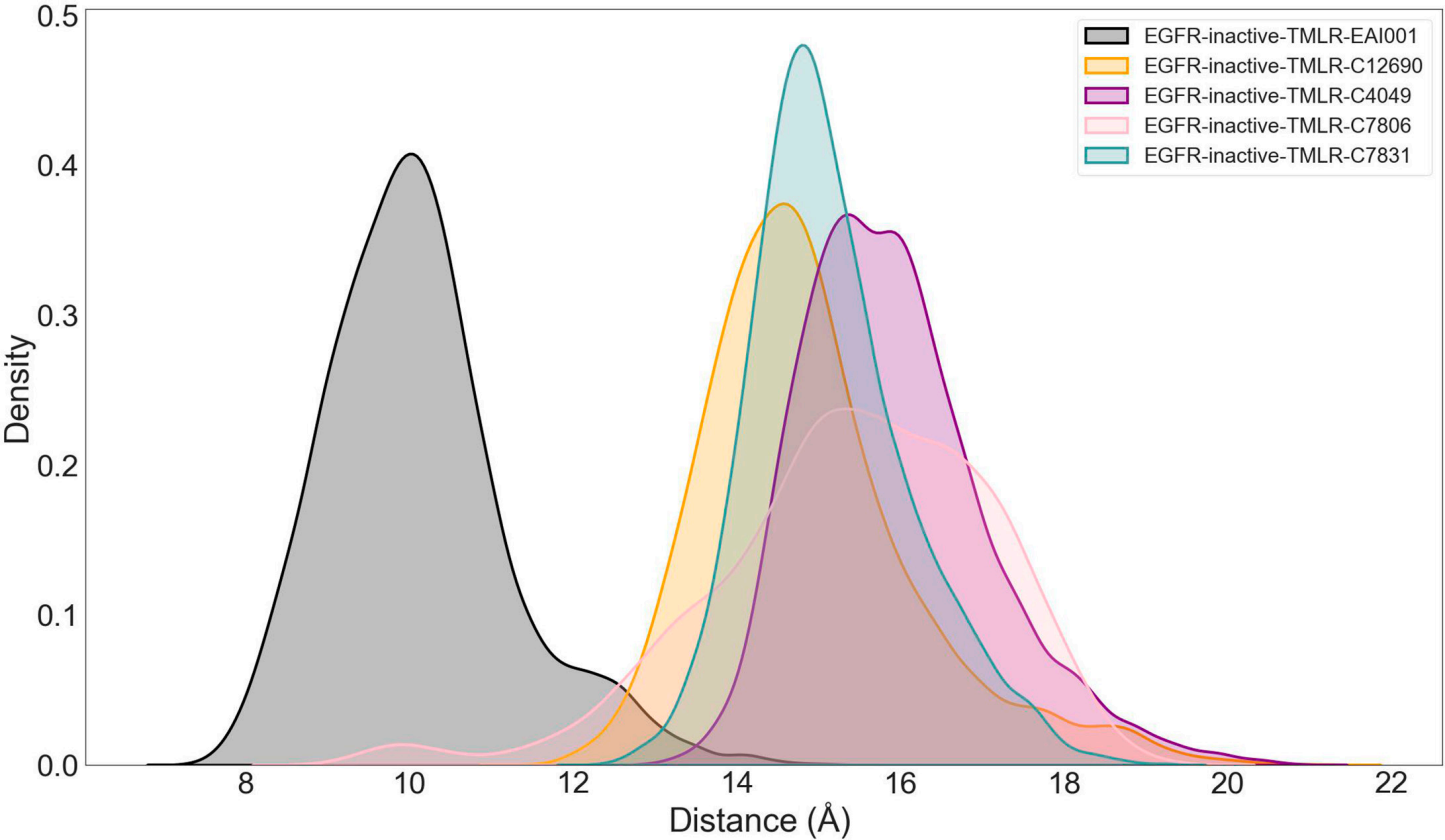
K745–E762 salt-bridge distance distributions for EGFR^L858R/T790M^ bound to EAI001 and the 4 candidate compounds (C7806, C4049, C12690, and C7831). Kernel density estimates of the distance between the NZ atom of K745 and the CD atom of E762 are shown for 100-ns simulations of apo-inactive EGFR^L858R/T790M^ in complex with EAI001 and the 5 compounds that most strongly shift the K745–E762 distance toward larger, inactive-like values. The remaining compounds are shown in Fig. S17.

We also analyzed the impact of the modulators on the K745–E762 distance, a key determinant of EGFR activation. Notably, all compounds exhibited a larger average distance (ranging from 11.8 to 15.9 Å) between salt-bridge-forming residues when bound to the inactive EGFR^L858R/T790M^ compared to EAI001-bound inactive EGFR^L858R/T790M^ during the 100-ns simulation. The compounds C7806, C4049, C7831, and C12690 showed the most substantial increases, followed by C4481, C19017, C19004, C11596, C7826, and C7819 (Fig. 7 and Fig. S17). These short 100-ns simulations suggest that the screened compounds hold promise as potential allosteric modulators of EGFR^L858R/T790M^ by stabilizing the inactive state. However, longer time-scale simulations will be required to fully characterize the long-term effects of these compounds on the EGFR conformation and their modulator mechanisms.

To further characterize binding-pocket interactions of the top candidates alongside EAI001, residue contact frequency and H-bond occupancy were calculated over the full 100-ns trajectories. Lys745 contacts all 4 compounds (C4481, C7831, C11596, and EAI001) at >40% occupancy. Asp855 and Met790 form the highest-occupancy H-bonds with C4481 (2.29% and 2.23%, respectively). Thr854 shows the highest H-bond occupancy with C7831 (~14.2%) (Fig. S18A and B).

Next, we calculated the relative binding free energies (Δ*G*^total^) of the compounds using MM/GBSA analysis (Table 3). All compounds showed higher binding affinities than EAI001 (−35.12 ± 2.98 kcal/mol), including the top 3: C11596 (−50.76 ± 3.97 kcal/mol), C4481 (−49.83 ± 2.91 kcal/mol), and C7831 (−49.92 ± 3.88 kcal/mol). These values represent relative binding-affinity rankings; absolute binding free energies would require entropic corrections. It is explicitly noted that the 100-ns simulations of the 10 candidate compounds are used for comparative ranking and initial assessment of allosteric pocket interactions, MM/GBSA binding affinity, and qualitative αC-helix and salt-bridge behavior, not for capturing complete inactive-to-active conformational transitions, which require microsecond-scale sampling. Table 4 outlines the categories used, including docking score, ML-based predicted score, αC-helix displacement, salt-bridge distance, and MM/GBSA scores. Although SG-ML-PLAP scores were used to prioritize the initial top 10 candidates, we also retained Vina scores in the final selection because the 2 methods capture complementary aspects of binding. Vina provides a physics-based assessment of pose quality and steric/enthalpic complementarity, whereas SG-ML-PLAP is trained to predict binding affinity from interaction fingerprints. Using both metrics together helps reduce false positives that might arise if either pose quality or predicted affinity was considered in isolation. Compounds were selected if they ranked within the top 5 in at least 3 of these categories, ensuring a comprehensive assessment of their potential. Taken together, these results indicate that compounds C4481, C7806, C4049, C11596, and C7831 are promising candidates for further development as allosteric modulators of EGFR^L858R/T790M^. They exhibit high binding affinities, induce substantial structural changes that favor the inactive conformation, and possess favorable drug-like properties.

**Table 3.** Molecular Mechanics Generalized Born Surface Area (MM/GBSA) analysis for calculating the binding affinity of EAI001 and top 10 compounds with EGFR^L858R/T790M^ after simulations

Name	VDW	EEL	EGB	ESURF	Δ*G* GAS	Δ*G* SOLV	Δ*G* TOTAL	SD
C11596	−68.35	−19.64	45.48	−8.25	−87.99	37.23	−50.76	3.97
C7831	−66.32	−35.91	60.01	−7.70	−102.23	52.31	−49.92	3.88
C4481	−69.80	−8.29	35.49	−7.27	−78.10	28.21	−49.83	2.91
C4049	−60.42	−50.05	69.13	−7.13	−110.48	62.0	−48.47	3.07
C7806	−66.0	−29.38	56.59	−8.10	−95.38	48.49	−46.89	3.62
C19005	−63.78	−14.65	38.84	−7.21	−78.43	31.62	−46.81	3.14
C7819	−59.16	−15.05	36.62	−7.52	−74.22	29.10	−45.12	4.38
C19017	−59.20	−12.19	37.96	−6.82	−71.36	31.14	−40.21	3.19
C12690	−52.66	−4.27	25.85	−6.58	−56.93	19.26	−37.66	2.90
C7826	−53.27	−9.75	32.20	−6.82	−63.03	25.38	−37.65	4.80
EAI001	−49.54	−17.52	37.99	−6.06	−67.06	31.93	−35.12	2.85

Abbreviations: VDW, van der Waals energy; EEL, electrostatic energy; EGB, generalized Born electrostatic solvation energy; ESURF, nonpolar solvation free energy; ΔG GAS, gas-phase energy contribution (VDW + EEL); ΔG SOLV, solvation free energy contribution (EGB + ESURF); ΔG TOTAL, total MM/GBSA binding free energy; SD, standard deviation.

**Table 4.** Multicriteria ranking of candidate allosteric modulators based on docking score, SG-ML-PLAP predicted affinity, C-helix displacement, K745–E762 salt-bridge distance, and MM/GBSA binding free energy. Compounds in bold achieved a top-5 rank in at least 3 of the 5 scoring criteria and were selected as lead allosteric candidates.

Name	SG-ML-PLAP score rank	αC-helix displacement rank	Salt-bridge distance rank	MM/GBSA score rank	Docking score rank
**C4481**	**1**	**6**	**5**	**3**	**4**
C19005	2	3	8	6	8
**C11596**	**3**	**8**	**7**	**1**	**2**
**C7806**	**4**	**1**	**1**	**5**	**7**
C7826	5	11	9	10	5
**C7831**	**6**	**4**	**3**	**2**	**9**
C7819	7	10	10	7	10
C12690	8	9	4	9	11
**C4049**	**9**	**2**	**2**	**4**	**3**
C19017	10	7	6	8	6
EAI001	11	5	11	11	1

## Discussion

EGFR kinase inhibitors play a crucial role in targeting mutant EGFR-driven cancers; however, the emergence of drug resistance remains a important challenge. Extensive research efforts have been dedicated to developing novel inhibitors against drug-resistant EGFR mutations. In this study, we employed all-atom MD simulations to investigate the conformational changes associated with the L858R/T790M double mutation in inactive EGFR and were able to capture key structural shifts that differentiate mutant EGFR from its wild-type counterpart. Our simulations revealed that wild-type EGFR in its inactive state adopts a conformation, characterized by a stabilized outward-positioned αC-helix and a potentially contracted A-loop. Upon introducing the L858R/T790M double mutation, a distinct shift toward an active-like conformation was observed, marked by the inward movement of the αC-helix and an extended A-loop. Although complete formation of the stable K745–E762 salt bridge was not observed for EGFR^L858R/T790M^, when simulations were started from the inactive conformation, our extended simulation analysis indicated a decrease in the distance between salt-bridge-forming residues upon mutation. This structural shift suggests that the L858R/T790M mutation shifts the conformational ensemble of the inactive kinase toward intermediate/active-like substates, potentially contributing to drug-resistance mechanisms. Furthermore, our study provides mechanistic insights into the allosteric inhibition of mutant EGFR by EAI001. Simulations of L858R/T790M mutant EGFR in complex with EAI001 revealed the adoption of an intermediate conformation, featuring a disordered αC-helix, a partially contracted A-loop, and a larger distance between salt-bridge-forming residues. These conformational changes indicate that in our simulations, EAI001 could not induce complete transition to the inactive state of EGFR^L858R/T790M^, with the potential for increased stabilization over extended simulation time. Overall, our simulations bridge this knowledge gap by elucidating how the driver/resistance mutation alters structural response of EGFR to allosteric inhibition. While this study employed the K745–E762 salt-bridge distance and the E762–D855 inter-residue distance as collective variables for 2D-FES analysis, additional activation metrics such as DFG φ/ψ dihedral classification and regulatory spine assembly were not computed and would provide a more complete multidimensional characterization of the activation landscape in future studies.

In search of novel and more effective allosteric inhibitors, we conducted virtual screening using the ChemDiv kinase allosteric inhibitor library. The docked complexes were reranked using SG-ML-PLAP, a recently developed ML-based scoring function. The top 10 compounds were selected based on their SG-ML-PLAP scores, and subsequently, 100-ns MD simulations were performed to assess their potential to induce the transition of EGFR^L858R/T790M^ to the inactive state by binding to the allosteric site. Δ*G*_bind_ calculations using MM/GBSA analysis further validated the high affinity of these compounds for the mutant kinase. We note that direct numerical comparison across various studies published earlier is complicated by differences in force-field parameterization, dielectric constant, entropy treatment, and snapshot extraction protocol. Our results are therefore interpreted as relative rankings rather than absolute predictions, and the top candidates (C4481, C7831, and C11596) are identified as priority compounds for future experimental validation [46–48]. We note, however, that longer microsecond-scale simulations will be required to fully confirm persistent pocket occupancy and assess compound off-rates. Among the screened molecules, our multicriteria analysis highlighted compounds C4481, C7806, C4049, C11596, and C7831 as having predicted allosteric binding profiles more favorable than EAI001, making them promising candidates for further investigation. Considering the scarcity of highly selective allosteric modulators for EGFR^L858R/T790M^, this study provides structural and conformational insight into potential high-affinity compounds that may serve as next-generation allosteric inhibitors. Future in vitro and in vivo studies will be required to validate their inhibitory efficacy and therapeutic potential. In summary, we have developed an in silico protocol for prioritizing allosteric modulators that modulate the conformational dynamics of flexible drug targets.

## Data Availability

The crystal structures used in this study are publicly available from the RCSB Protein Data Bank (https://www.rcsb.org/). PDB and SMILES strings for all 10 candidate allosteric inhibitors identified through virtual screening are provided as a supplementary ZIP file accompanying this article. Key MD trajectory data supporting the findings of this study are available upon reasonable request.

## Supplementary Materials

Supplementary 1 Tables S1 to S3 Figs. S1 to S18 Top docked structure coordinates
