# Supplementary material for: Deciphering the Structural Basis of Allosteric Inhibition of Mutant Epidermal Growth Factor Receptor and Identification of Novel Inhibitors
Source: Comput Struct Biotechnol J. 2026 May 27;35(1):0118. doi: 10.34133/csbj.0118 (PMC13213074; doi:10.34133/csbj.0118)
Supplement: Supplementary 1 — Tables S1 to S3 Figs. S1 to S18 Top docked structure coordinates [file csbj.0118.f1.zip › Sapna_CSBJ_Supplementary_material_clean.docx]

**Design of Allosteric Inhibitors for Mutant EGFR by Combined use of Machine Learning and Molecular Dynamics Simulations**

**Sapna Pal^1^ and Debasisa Mohanty^1*^**

**Bioinformatics Center**

**BRIC-National Institute of Immunology**

**Aruna Asaf Ali Marg, New Delhi – 110067, India**

^1^Bioinformatics Center, BRIC-National Institute of Immunology, New Delhi, India

**^*^Address correspondence to:**

Debasisa Mohanty, Bioinformatics Center, BRIC- National Institute of Immunology, Aruna Asaf Ali Marg, New Delhi-110067, India; Tel: +91-11-26703749; Fax: +91-11-26742125; E-Mail: deb@nii.ac.in

**Supplementary Table S1**. Effect of electrostatic cutoff on the K745–E762 distance in inactive EGFR systems. Values are mean ± standard deviation (Å) of the K745–E762 distance over 700ns trajectories simulated with 8, 13, or 16 Å nonbonded cutoffs.

| **System** | **8 Å cutoff (Å)** | **13 Å cutoff (Å)** | **16 Å cutoff (Å)** |
| --- | --- | --- | --- |
| Apo inactive EGFRWild | 14.59 ± 1.36 | 15.05 ± 1.39 | 14.07 ± 1.69 |
| Apo inactive EGFRT790M/L858R | 11.60 ± 1.34 | 12.10 ± 1.76 | 11.38 ± 1.40 |
| EAI001 inactive EGFRT790M/L858R | 12.35 ± 1.56 | 9.45 ± 0.78 | 11.51 ± 1.74 |

**Supplementary Table S2.** Time‑resolved populations of K745–E762 distance regimes in the inactive EAI001‑bound EGFR^L858R/T790M^ simulation. Frames from the 0–2 µs and 8–10 µs windows were classified as inactive‑like (K745–E762 > 13 Å), intermediate‑like (7–13 Å), or active‑like (< 7 Å), and the percentage of frames in each state is reported together with 95% binomial confidence intervals.

| **State** | **0–2 µs %** | **(95% CI)** | **8–10 µs %** | **(95% CI)** |
| --- | --- | --- | --- | --- |
| Inactive-like >13 Å | 33.6% | (33.4–33.8%) | 61.9% | (61.7–62.2%) |
| Intermediate 7–13 Å | 66.4% | (66.2–66.6%) | 38.1% | (37.8–38.3%) |
| Active-like <7 Å | 0.0% | (0.0–0.0%) | 0% | (0.0–0.0%) |

**Supplementary Table S3.** Percentage occupancies of inactive‑like, intermediate‑like, and intermediate/active‑like conformations for inactive EGFR systems. States were defined using the K745–E762 distance and E762–D855 inter-residue distance as a directional reporter of αC-helix positioning as collective variables (see Methods), and values are reported as mean ± standard error over the trajectories.

| **System** | **Inactive‑like (%)** | **Intermediate‑like (%)** | **Intermediate/Active‑like (%)** |
| --- | --- | --- | --- |
| Apo inactive EGFRWild | 80.5 ± 0.2 | 19.5 ± 0.2 | 0.0 ± 0.0 |
| Apo inactive EGFRT790M/L858R | 0.1 ± 0.0 | 99.9 ± 0.0 | 0.0 ± 0.0 |
| EAI001 inactive EGFRT790M/L858R | 15.6 ± 0.2 | 84.4 ± 0.2 | 0.0 ± 0.0 |


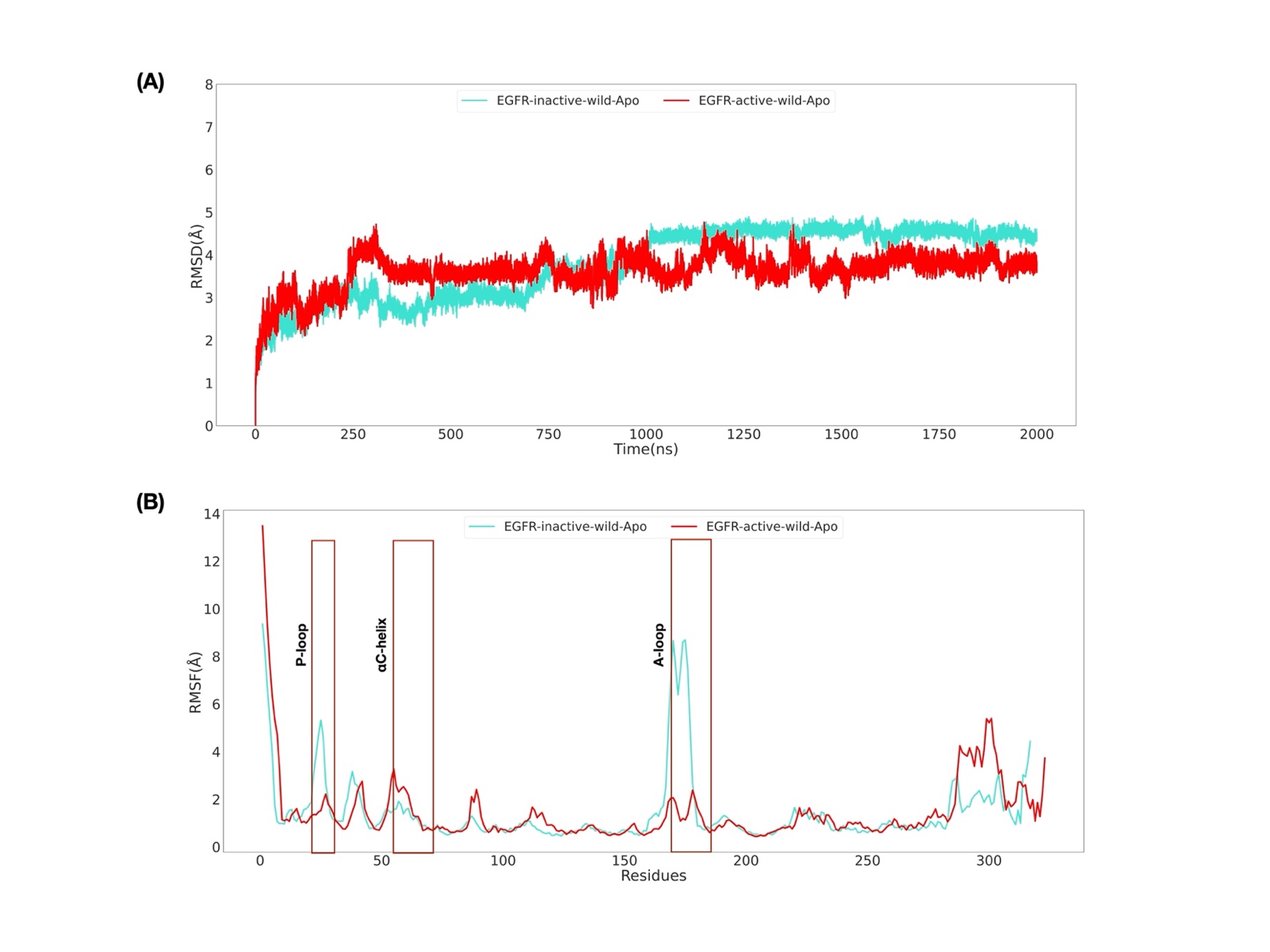


**Supplementary Figure S1.** RMSD (A) and RMSF (B) plot of active and inactive wild-type apo-EGFR upon 2µs simulation using 16Å cutoff. ***Note:** Residue numbering in this figure reflects the modelled EGFR kinase domain (residues 1-317), which corresponds to residues beginning at position 698 of the full-length EGFR protein.

^
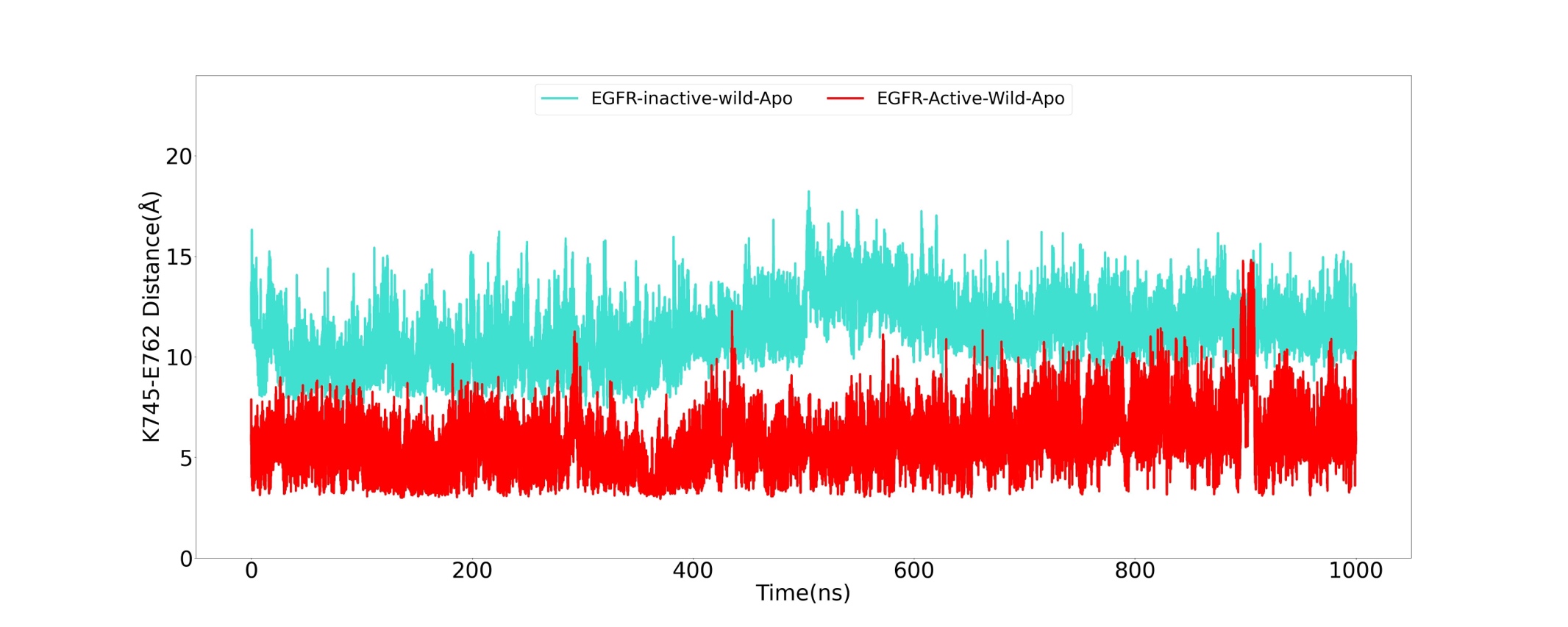
^

**Supplementary Figure S2.** Time series plot of the distance between NZ and CD atom of K745 and E762 salt bridge forming residues, respectively, for the simulation done using 13Å a non-bonded cutoff distance. Distance calculated for the conformations observed during 1 µs simulations of active and inactive wild-type apo-EGFR.


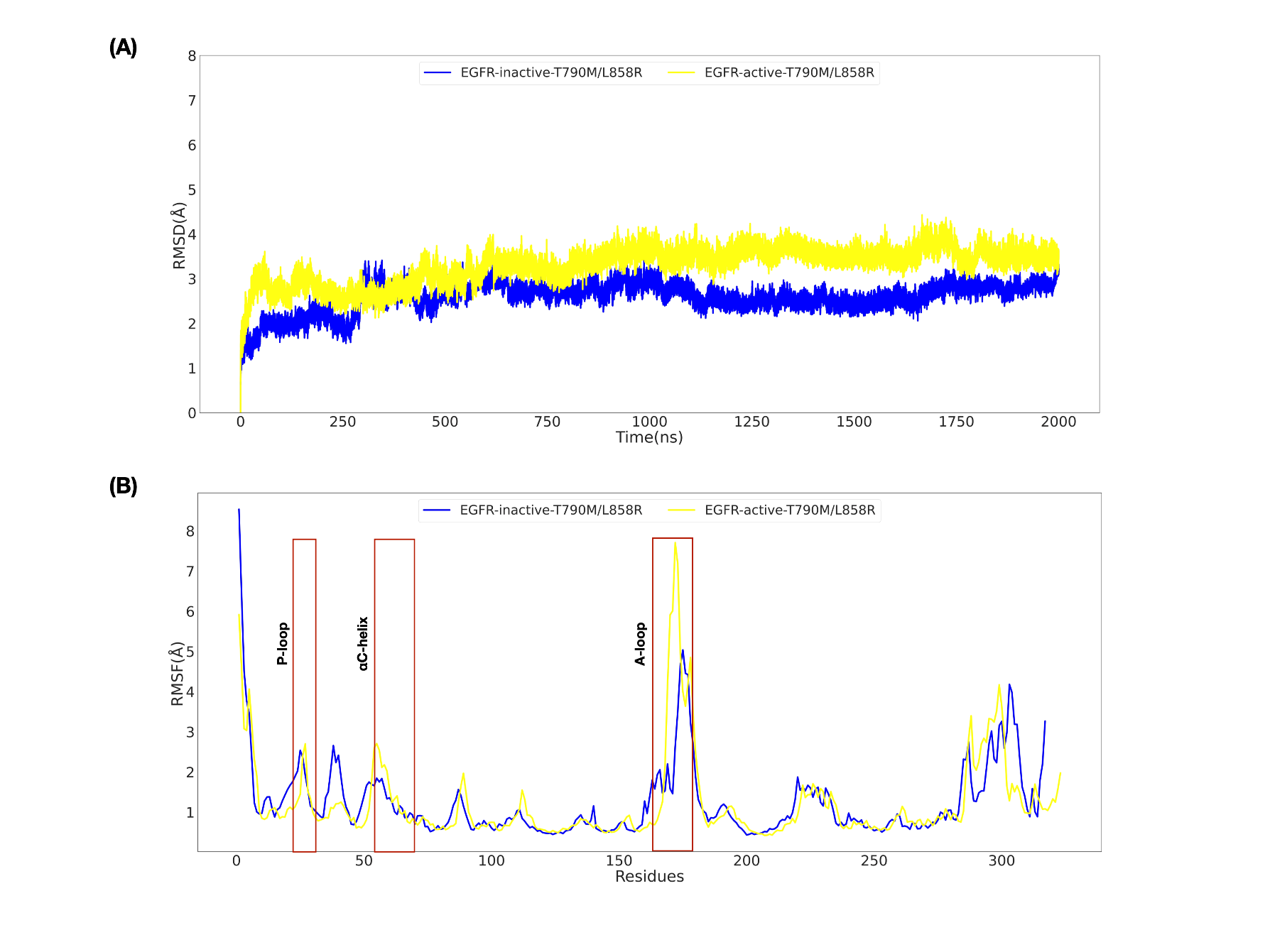


**Supplementary Figure S3.** RMSD (A) and RMSF (B) plot of active and inactive apo-EGFR^L858R/T790M^ upon 2µs simulation using 16Å cutoff. ***Note:** Residue numbering in this figure reflects the modelled EGFR kinase domain (residues 1-317), which corresponds to residues beginning at position 698 of the full-length EGFR protein.


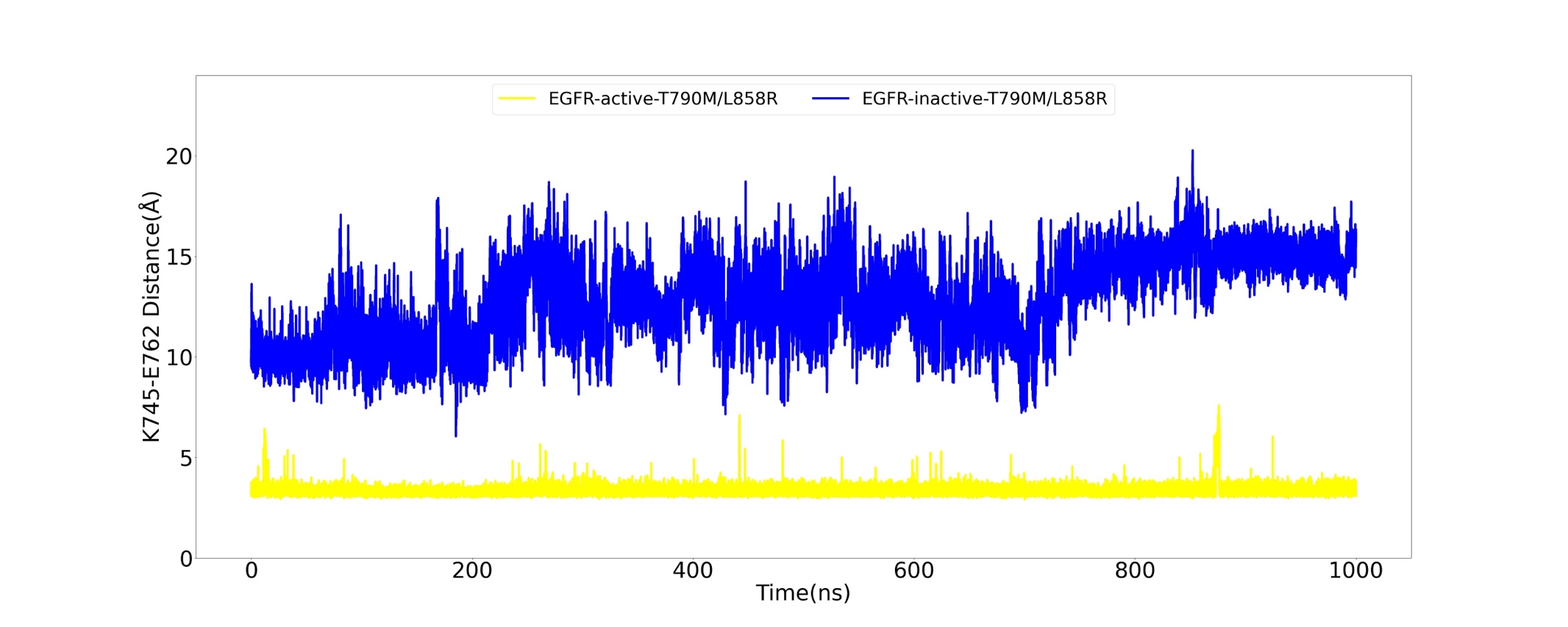


**Supplementary Figure S4.** Time series plot of the distance between NZ and CD atom of K745 and E762 salt bridge forming residues, respectively, for the simulation done using 13Å, a non-bonded cutoff distance. Distance calculated for the conformations observed during 1 µs simulations of active and inactive apo-EGFR^L858R/T790M^.


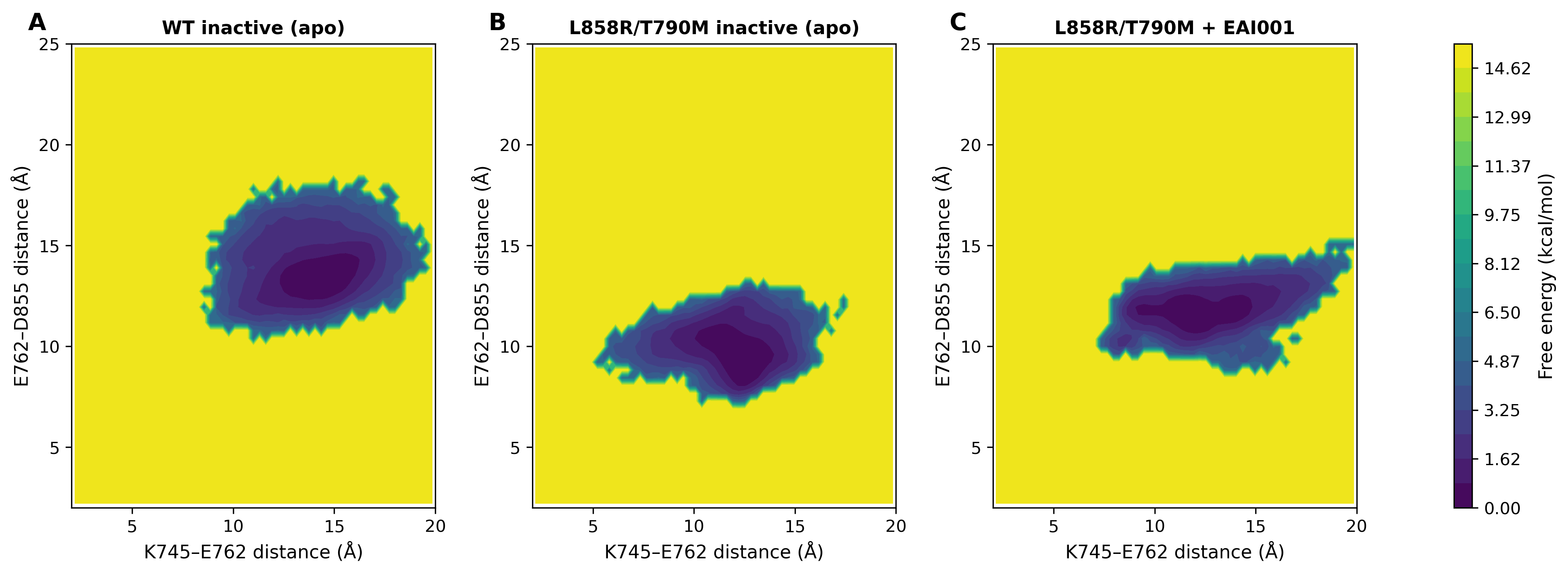


**Supplementary Figure S5.** Two-dimensional free energy surfaces (FES) for inactive EGFR systems. The FES was computed using the K745–E762 salt‑bridge distance and E762–D855 inter-residue distance as a directional reporter of αC-helix positioning as collective variables for three simulation systems. The color scale indicates the relative free energy in kcal/mol, with darker regions representing more populated (lower free energy) conformational states. Distinct conformational basins across the three systems reflect differences in K745–E762 distance preferences and αC‑helix positioning.


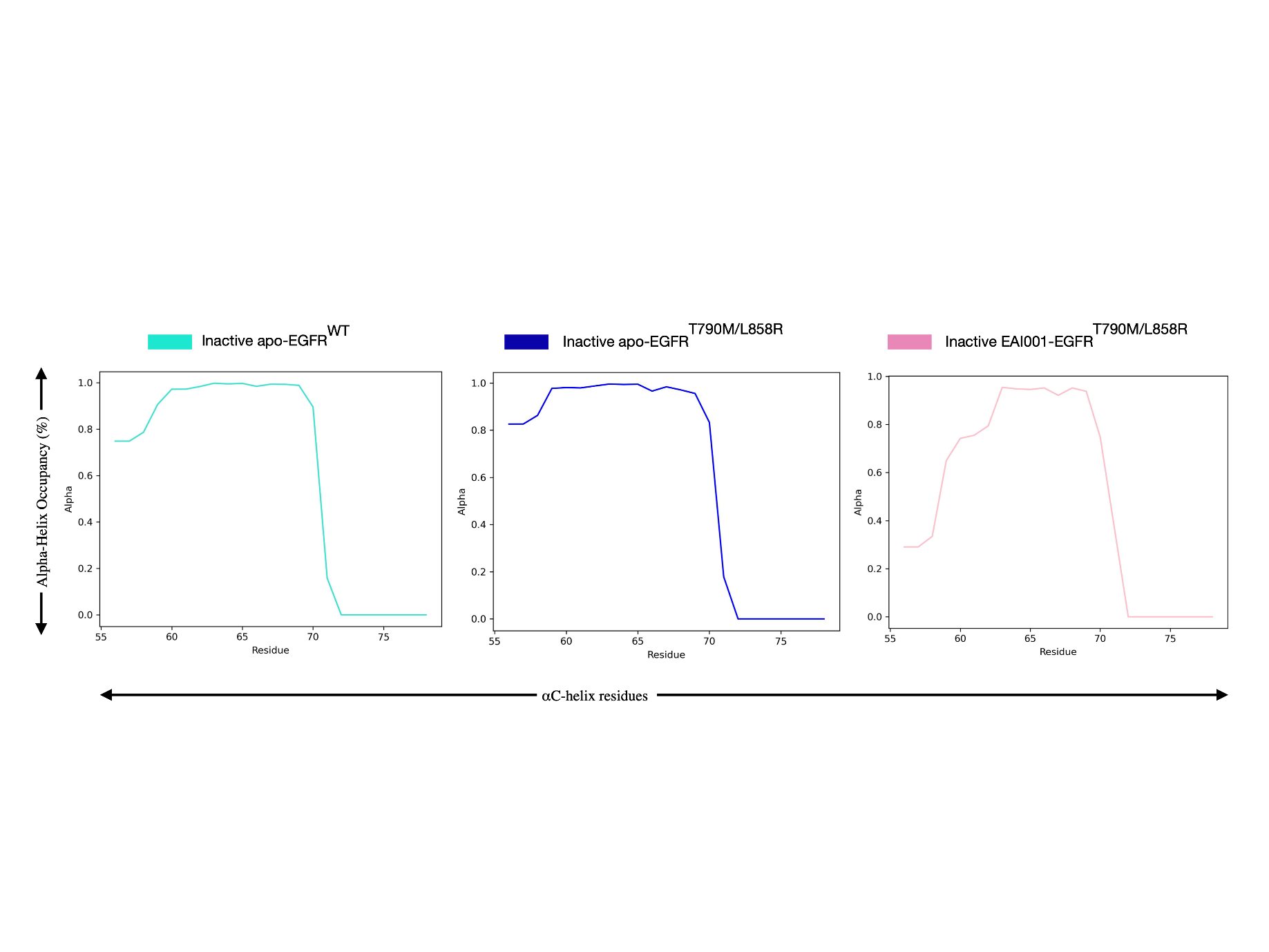


**Supplementary Figure S6.** Secondary structure analysis: Alpha-helix occupancy of αC-helix residues. The plot displays the percentage of simulation time that each αC-helix residue spends in an alpha-helical conformation across 2 µs simulations for inactive apo-EGFR^Wild^, apo−EGFR^L858R/T790M^, and EAI001−EGFR^L858R/T790M^.


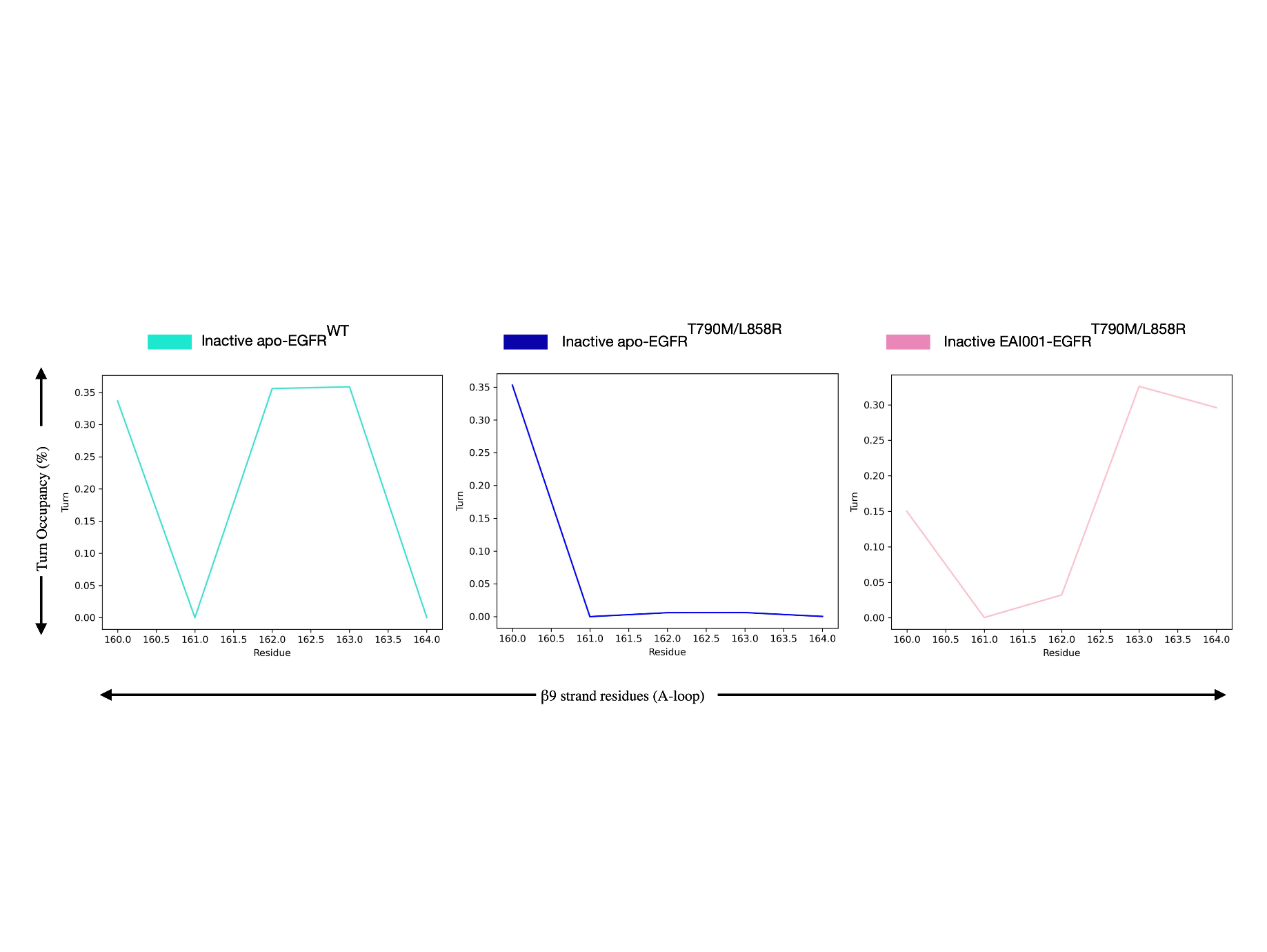


**Supplementary Figure S7.** Secondary structure analysis: Turns occupancy of β9 strand residues of the A-loop. The plot displays the percentage of simulation time each β9 strand residue of the A-loop spends in a turn conformation across 2 µs simulations for inactive apo-EGFR^Wild^, apo−EGFR^L858R/T790M^, and EAI001−EGFR^L858R/T790M^.


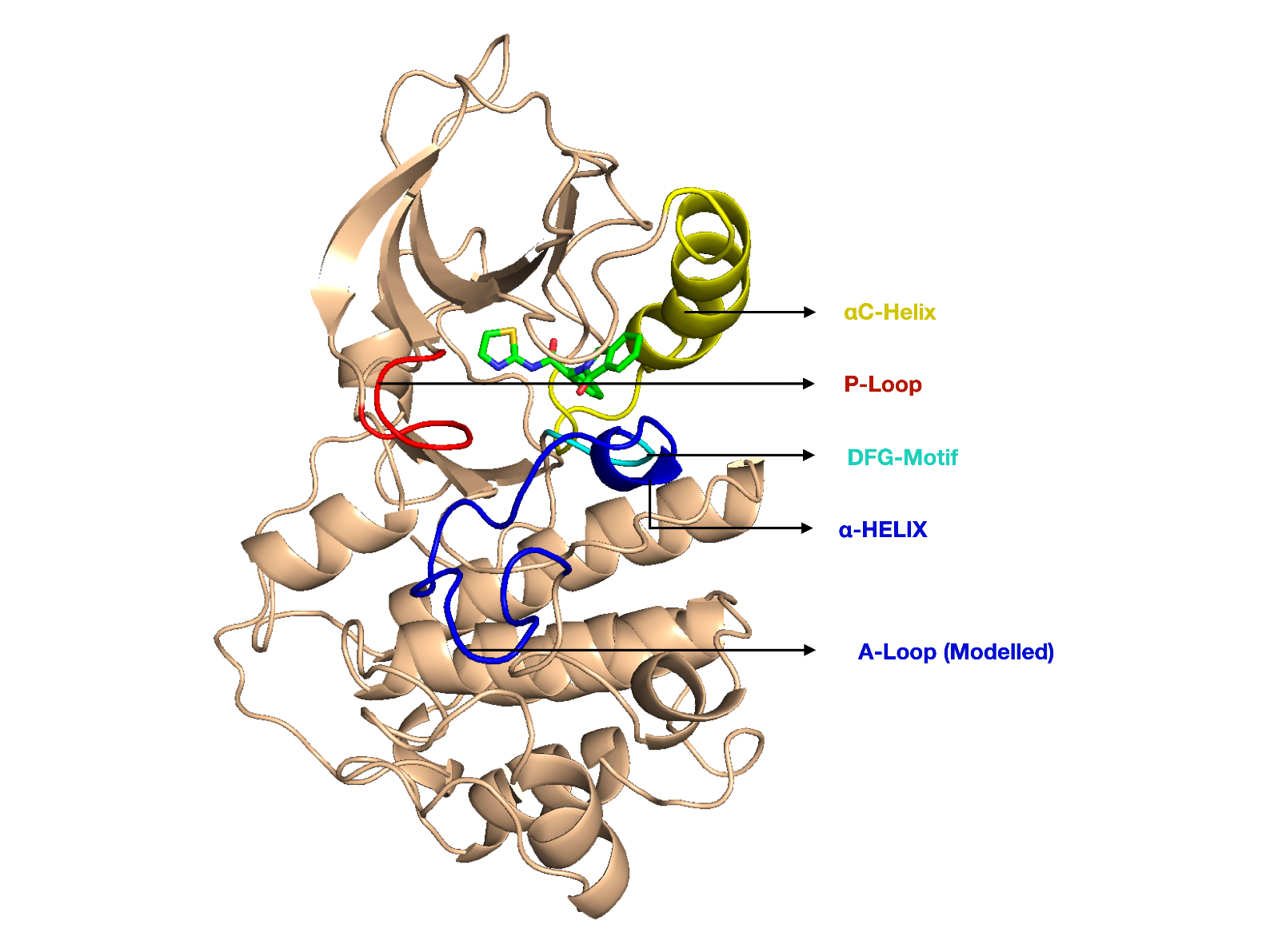


**Supplementary Figure S8.** Conversion of the β9 strand residues into an α-helix at the N-terminal of the A-loop after 8.5 µs.


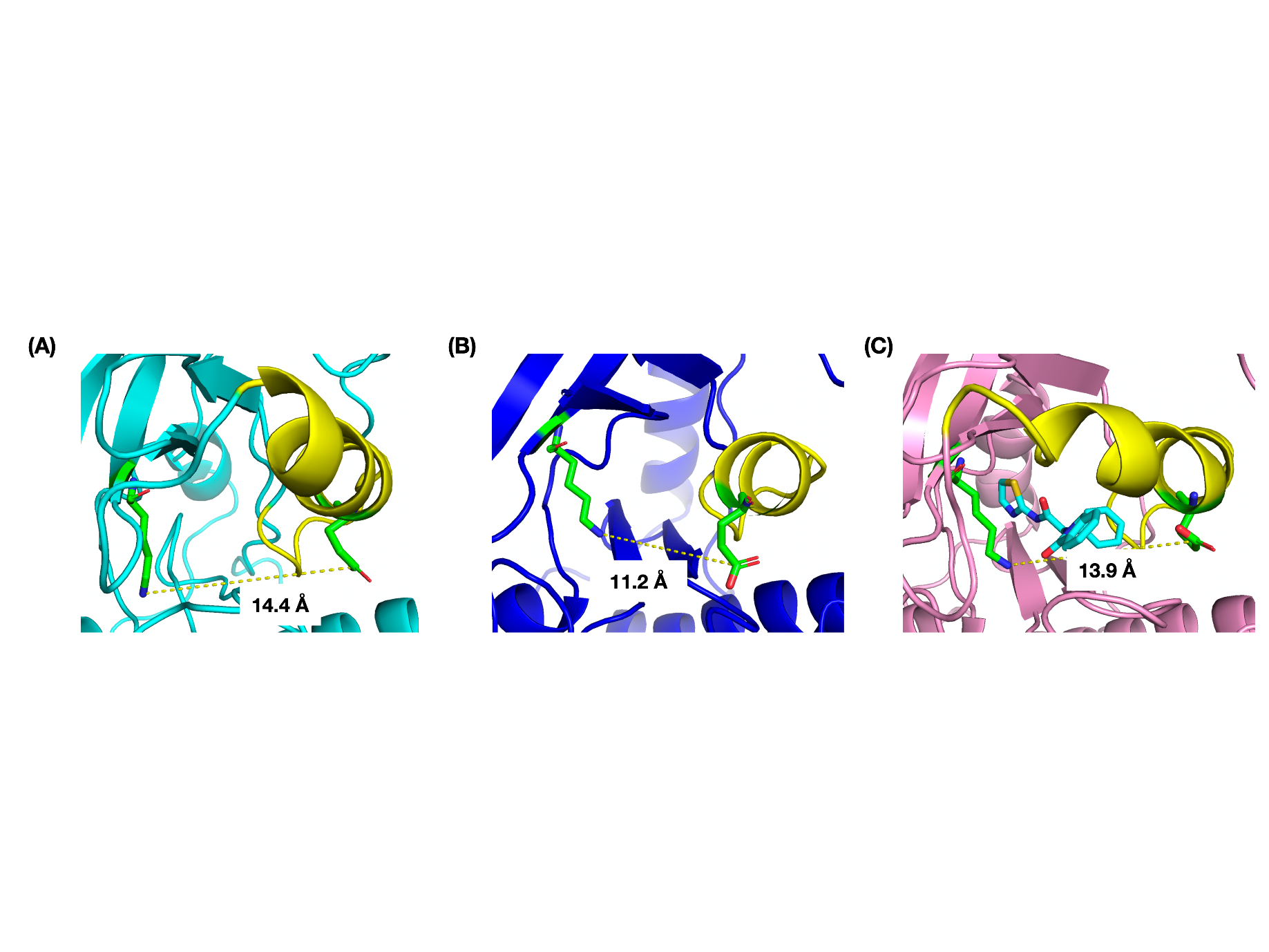


**Supplementary Figure S9.** Salt bridge distances between K745(NZ-atom) and E762(CD-atom) observed in representative conformations extracted from highly populated clusters in the PCA plot (Panels A, B, and C of Figure 4 for inactive apo-EGFR^Wild^, apo−EGFR^L858R/T790M^, and EAI001−EGFR^L858R/T790M^.


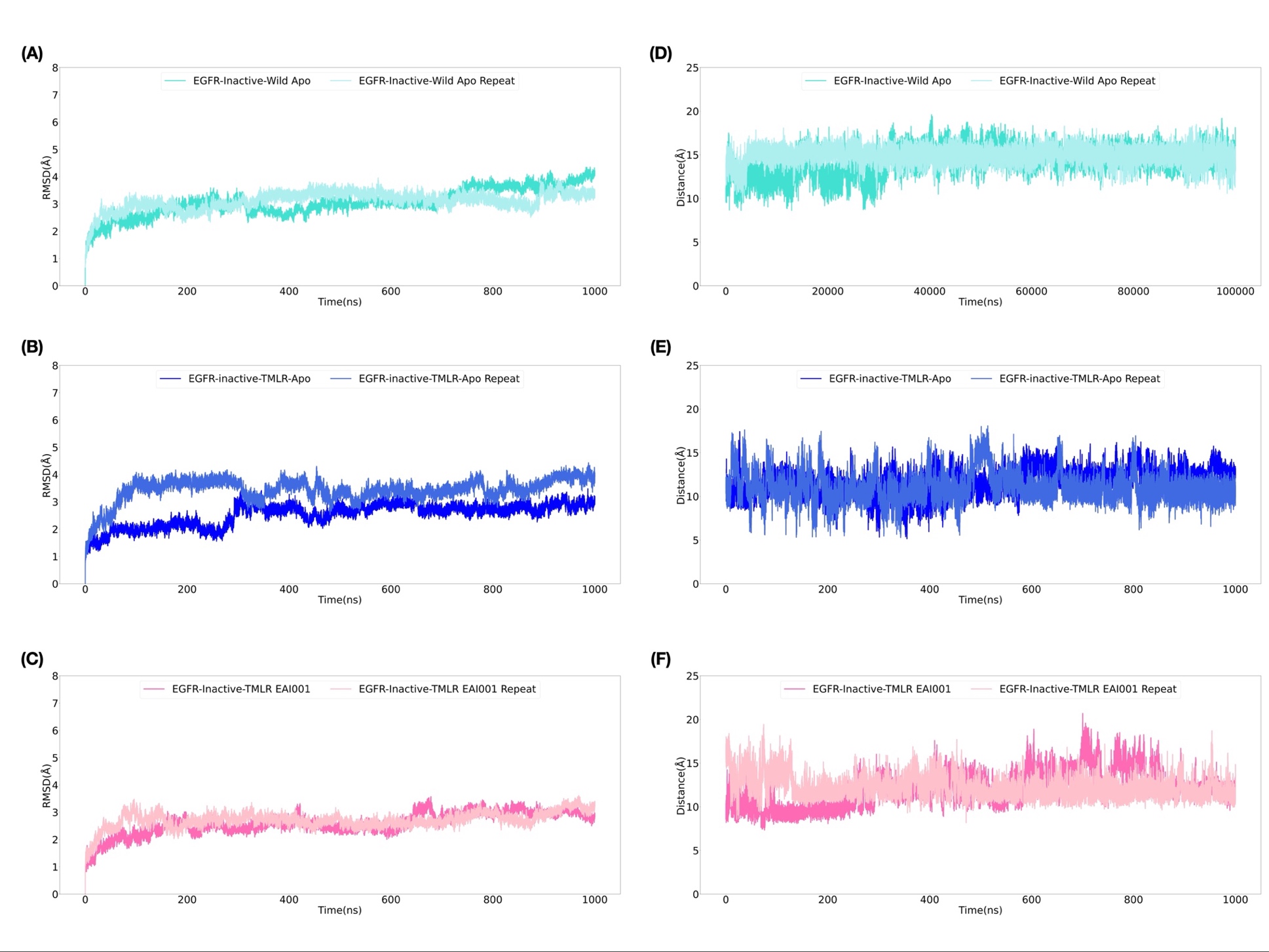


**Supplementary Figure S10**. Replicate simulations of inactive EGFR systems. (A–C) Backbone RMSD time series for apo‑inactive EGFR^Wild^ (cyan), apo‑inactive EGFR^L858R/T790M^ (blue), and EAI001‑bound EGFR^L858R/T790M^ (pink) and their corresponding 1 µs replicate trajectories. (D–F) Time series of the distance between the NZ atom of K745 and the CD atom of E762 for the same systems and replicates. The replicate trajectories closely follow the primary runs and do not reveal additional slow transitions, indicating that the observed RMSD and K745–E762 distance trends are reproducible over repeated simulations of this length.


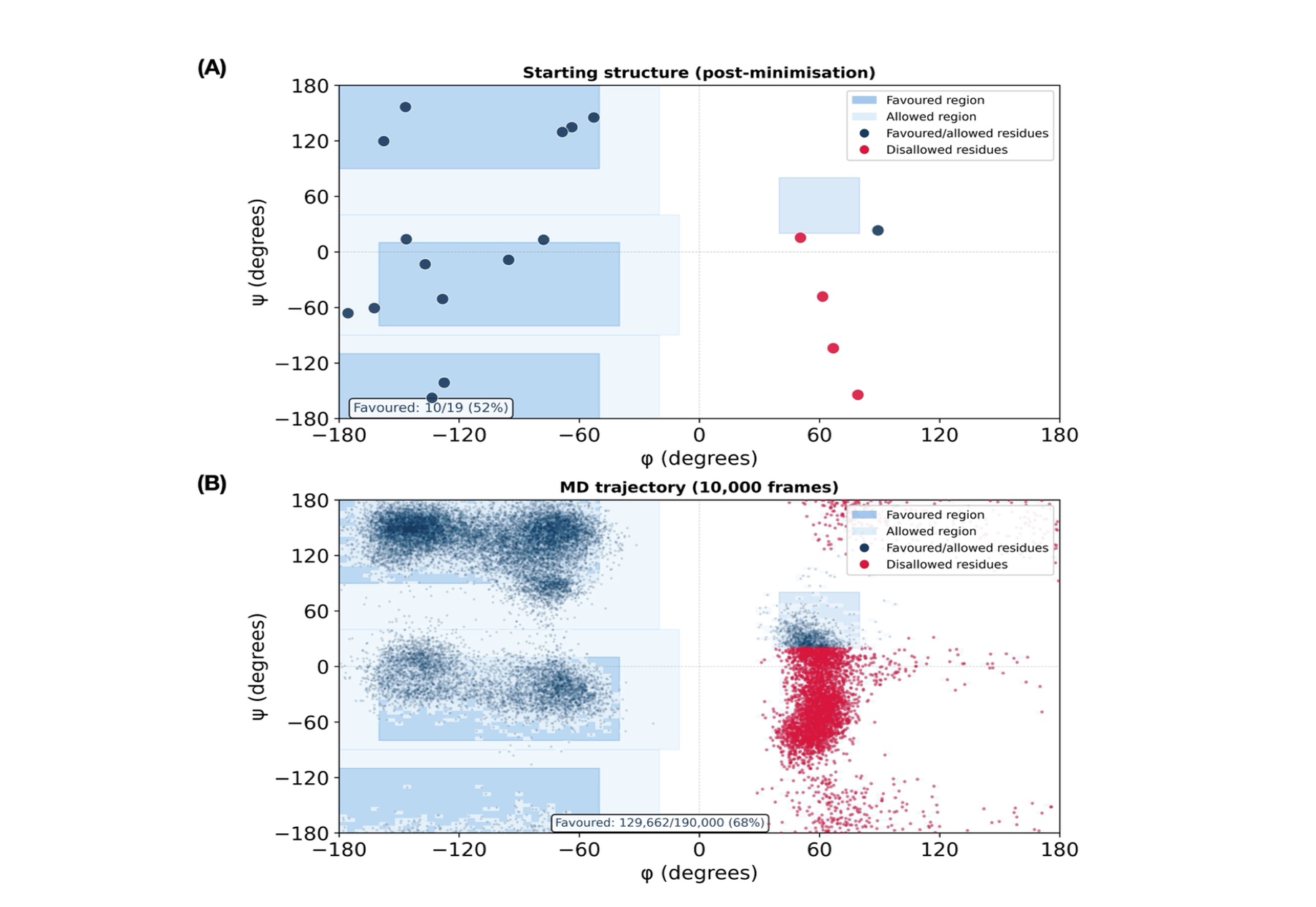


**Supplementary Figure S11.** Ramachandran analysis of the modelled A-loop (residues 855–877) in EGFR^L858R/T790M. (A) Ramachandran plot of the 19 A-loop residues in the post-minimization starting structure, with 52% (10/19) of residues falling in favored regions (dark green) and four residues (K860, A864, E866, and K875) in the disallowed region (red). (B) Ramachandran plot constructed from 10,000 frames of the 100 ns simulation trajectory (190,000 backbone dihedral angle pairs total), showing that 68% (129,662/190,000) of A-loop dihedral pairs occupy favored regions, indicating progressive relaxation of initially strained residues over the course of the simulation.

**
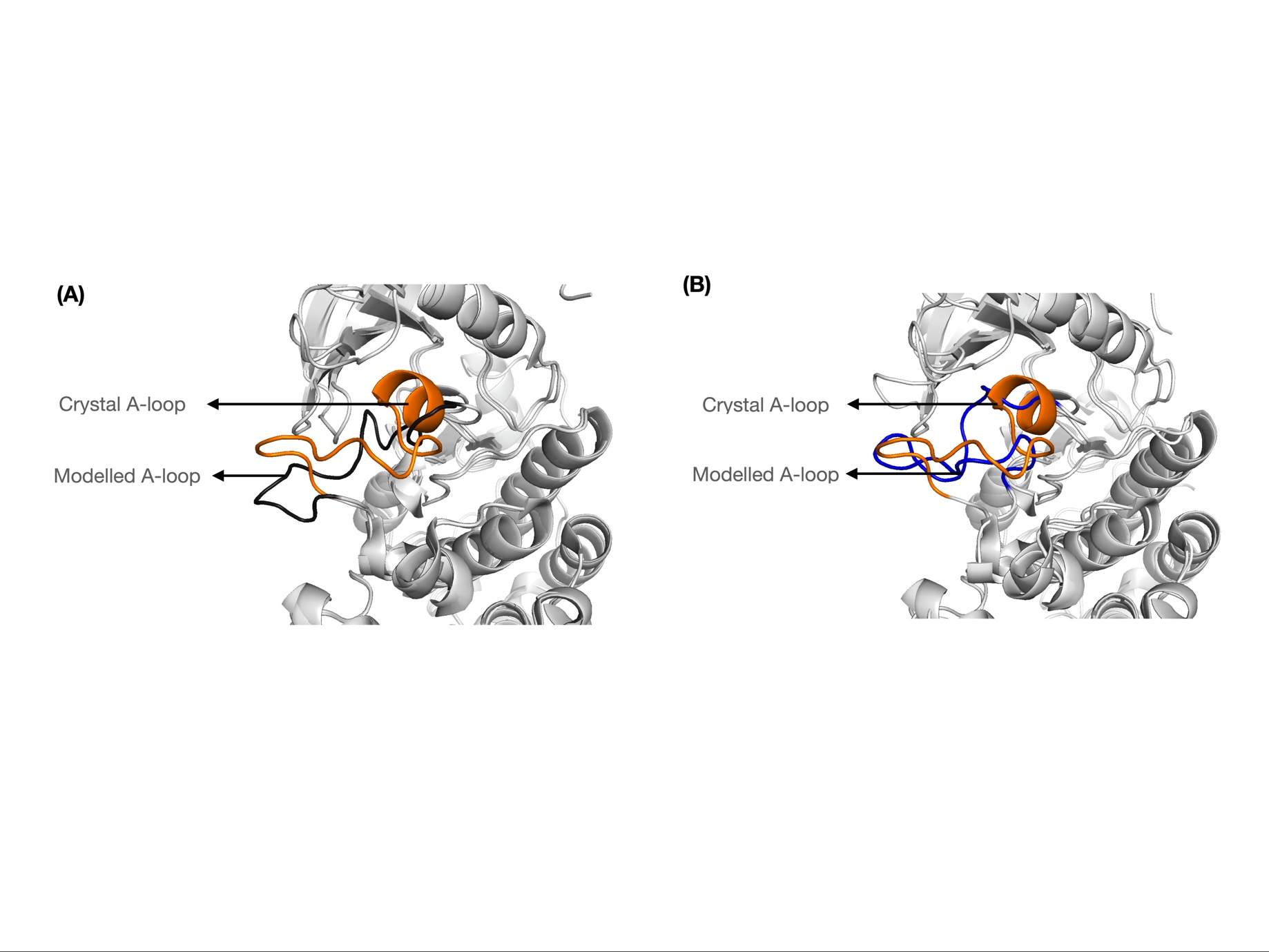
**

**Supplementary Figure S12.** Structural evolution of the modelled A-loop (residues 855–877) during simulation. (A) Overlay of the MODELLER-built A-loop (orange) (PDB ID: 5D41) with the reference inactive-state A-loop conformation (PDB ID: 5HJO) from the apo-inactive EGFR structure (black), showing that the modelled loop adopts a broadly similar fold without deviating into an unphysical conformation. (B) Post-100 ns A-loop position (blue) overlaid on the inactive-state reference (black), confirming that the loop stabilises in a conformation proximal to the inactive-state template region rather than drifting away throughout the simulation.


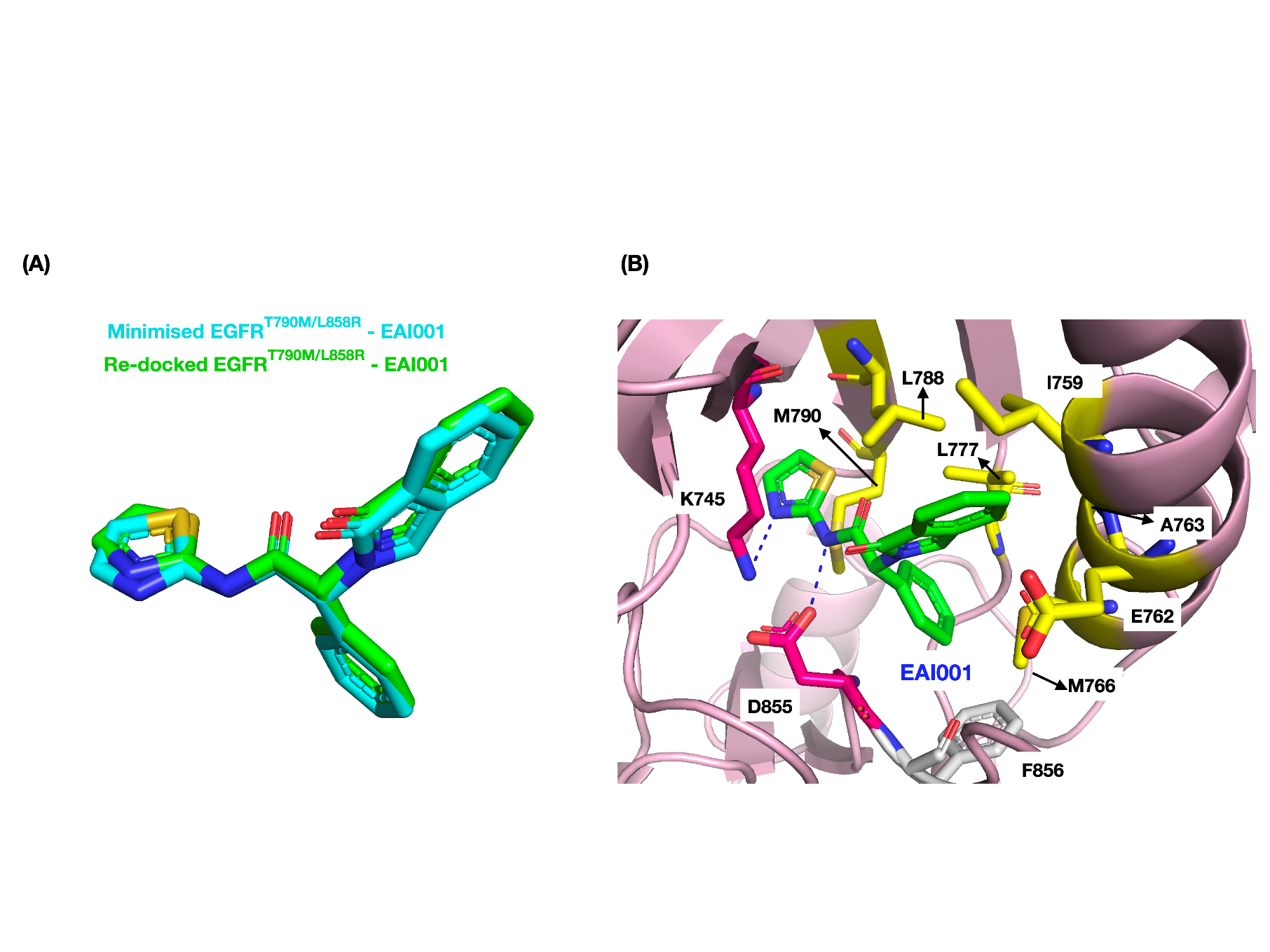


**Supplementary Figure S13.** Validation of docking parameters via re-docking of minimised EGFR^L858R/T790M^ in complex with EAI001 inhibitor. Superimposed image of minimised and re-docked structure of EAI001 on the allosteric pocket of EGFR^L858R/T790M^ (A). Hydrophobic (yellow) and hydrogen bond (pink) interacting residue of EGFR^L858R/T790M^ allosteric pocket with the re-docked ligand (B).


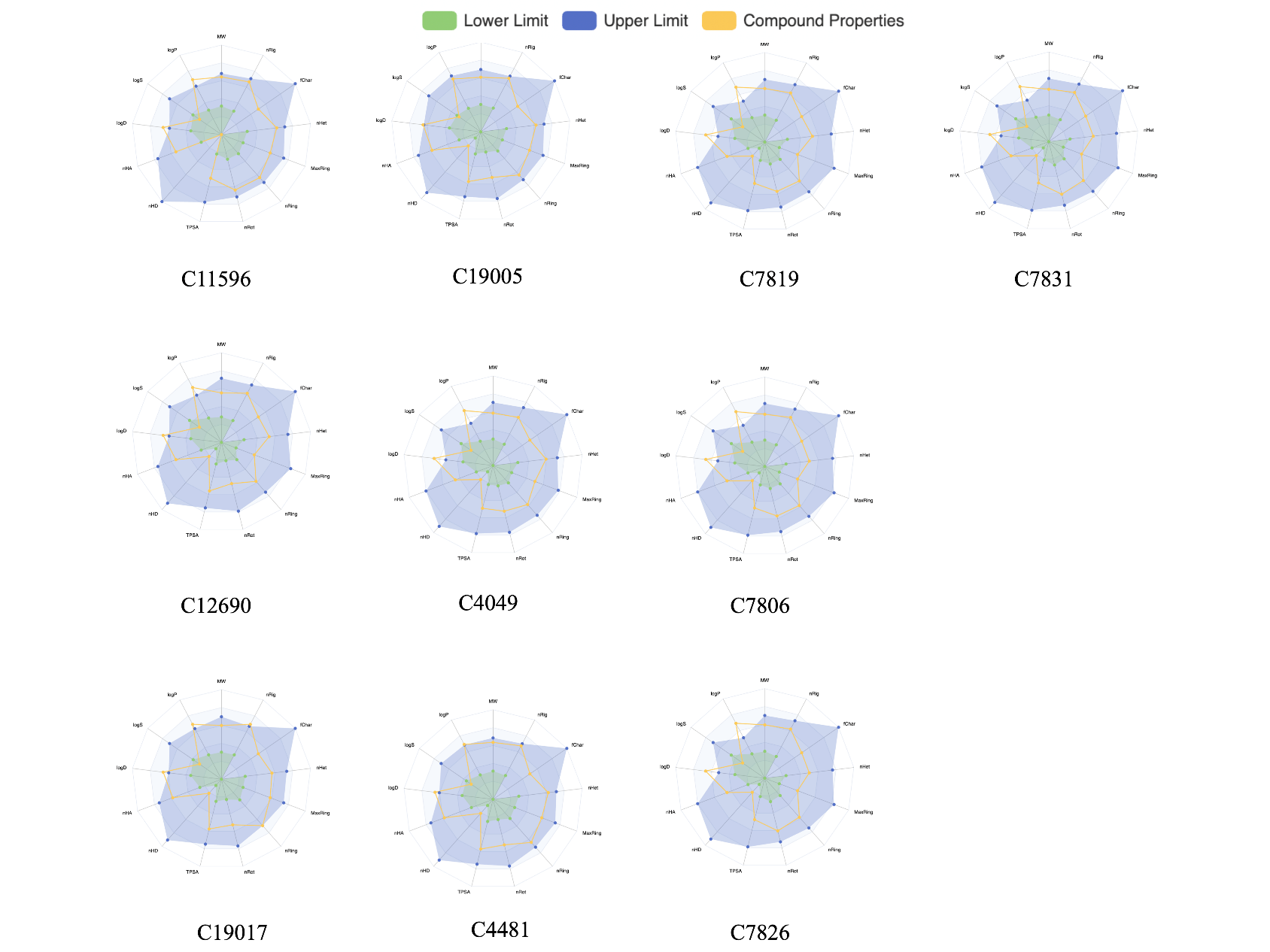


**Supplementary Figure S14.** Radar chart of drug likeliness and physicochemical properties of the top ten selected drugs predicted using the ADMETlab 3.0 software.


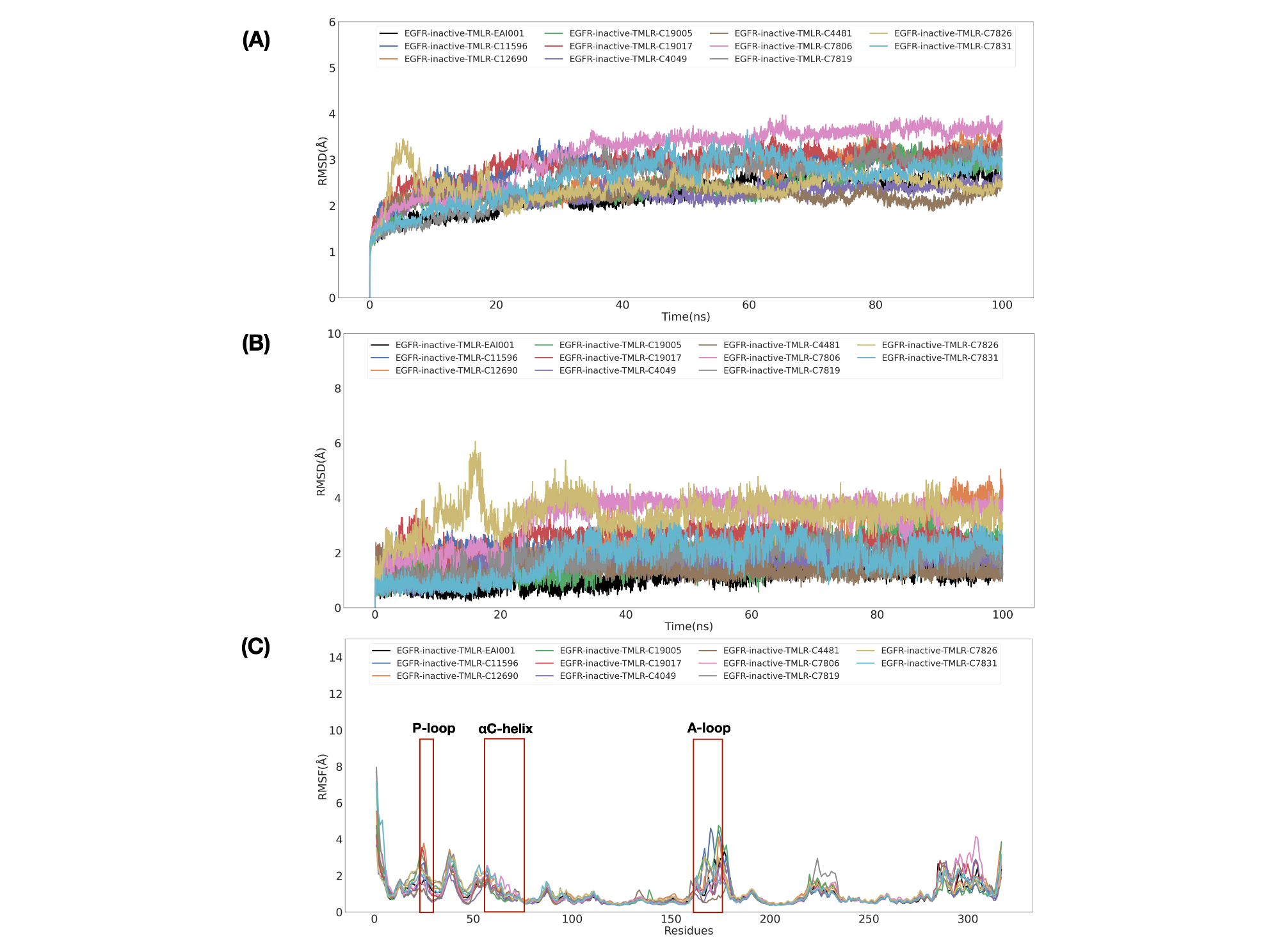


**Supplementary Figure S15.** Trajectory analysis of simulations of top ten compounds with EGFR^L858R/T790M^. RMSD plot of EGFR^L858R/T790M^ when simulated with EAI001 and selected compounds (A). RMSD plot EAI001 and selected compounds after the simulation (B). RMSF plot of EGFR^L858R/T790M^ residues after the simulation (C). ***Note:** Residue numbering in (**C**) reflects the modelled EGFR kinase domain (residues 1-317), which corresponds to residues beginning at position 698 of the full-length EGFR protein.


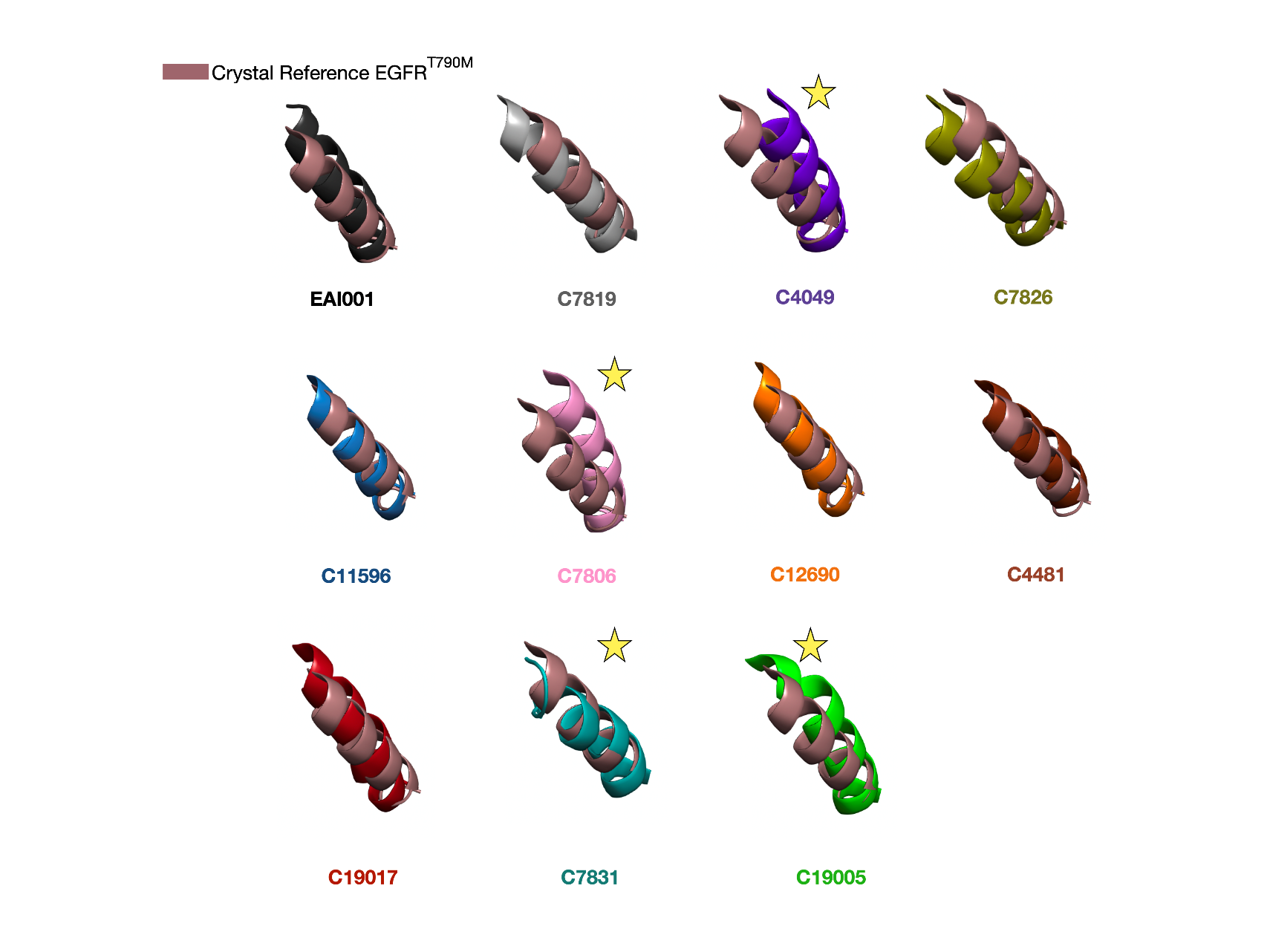


**Supplementary Figure S16.** Superimposition of the EGFR^L858R/T790M^ kinase conformations showing αC-helix (taken at the end of 100ns simulation with top ten compounds) onto the inactive EGFR crystal structure (PDB ID: 5D41) taken as a reference.


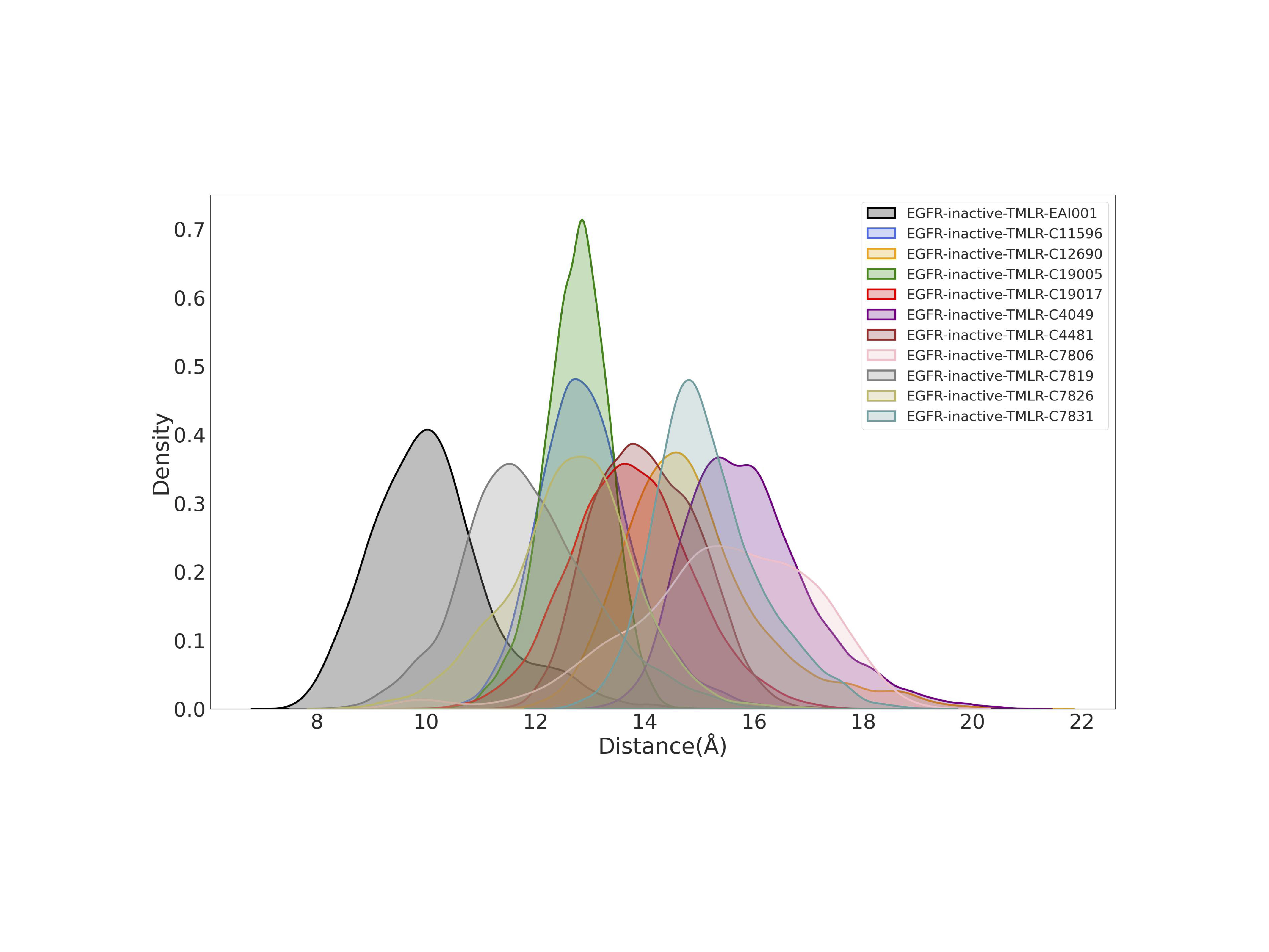


**Supplementary Figure S17.** K745–E762 salt‑bridge distance distributions for EGFR^L858R/T790M^ bound to EAI001 and the 10 candidate compounds. Kernel density estimates of the distance between the NZ atom of K745 and the CD atom of E762 are shown for 100 ns simulations of apo‑inactive EGFR^L858R/T790M^ in complex with EAI001 and the ten compounds.


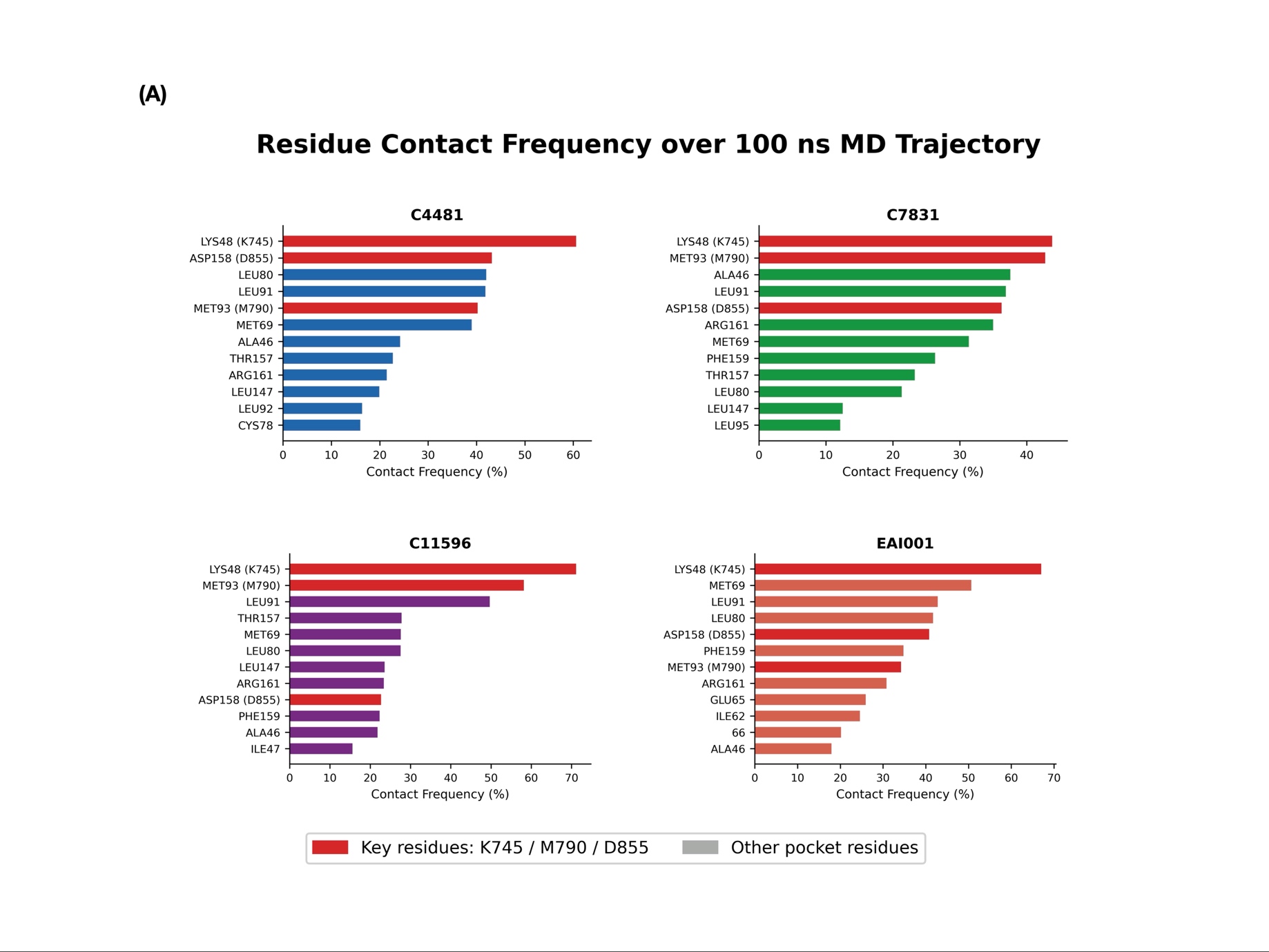


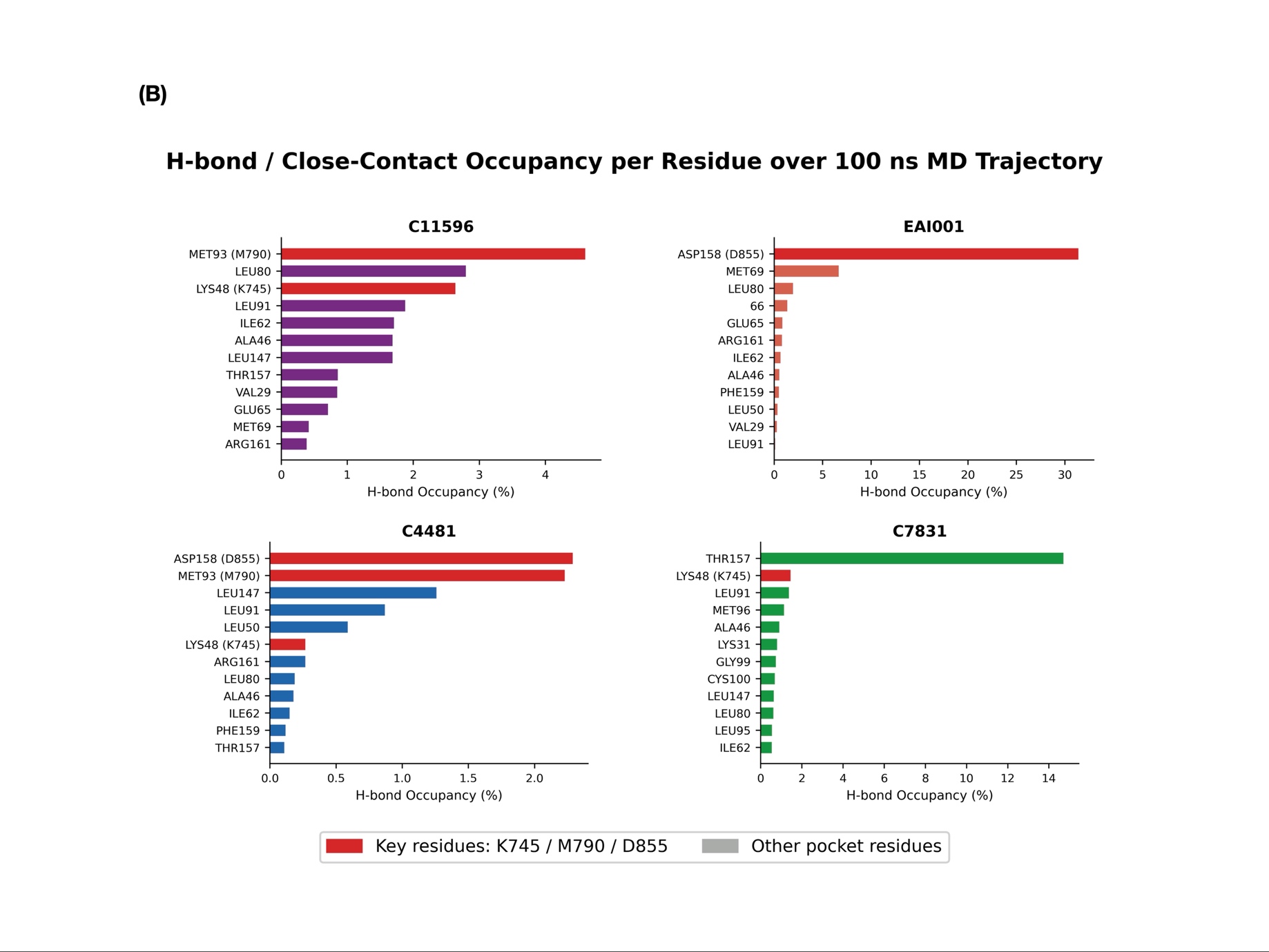


**Supplementary Figure S18.** Residue-level interaction analysis of top virtual screening candidates and EAI001 in the EGFR^L858R/T790M^ binding pocket over 100 ns MD simulations. (A) Residue contact frequency (%) for C4481, C7831, C11596, and EAI001, showing that Lys745 contacts all four compounds at >40% occupancy. (B) Hydrogen bond occupancy (%) between each compound and key binding pocket residues. Asp855 and Met790 show the highest H-bond occupancy with C4481 (2.29% and 2.23%, respectively), while Thr854 (renumbered as THR157) exhibits the highest H-bond occupancy with C7831 (~14.2%). Contact frequency and H-bond occupancy were calculated over all frames of the full 100 ns trajectory for each system.

Note: Residue labels in the figure reflect the internal numbering of the modelled EGFR kinase domain (residues 1–317, beginning at full-length position 698). The canonical EGFR residue equivalents are: LYS48 = Lys745, GLU65 = Glu762, MET93 = Met790, ASP158 = Asp855, THR157 = Thr854. All residues are referenced by their canonical numbers in the main text.
